# Dynamic evolution of the heterochromatin sensing histone demethylase IBM1

**DOI:** 10.1371/journal.pgen.1011358

**Published:** 2024-07-11

**Authors:** Yinwen Zhang, Hosung Jang, Ziliang Luo, Yinxin Dong, Yangyang Xu, Yamini Kantamneni, Robert J. Schmitz

**Affiliations:** Department of Genetics, University of Georgia, Athens, Georgia, United States of America; Gregor Mendel Institute of Molecular Plant Biology, AUSTRIA

## Abstract

Heterochromatin is critical for maintaining genome stability, especially in flowering plants, where it relies on a feedback loop involving the H3K9 methyltransferase, KRYPTONITE (KYP), and the DNA methyltransferase CHROMOMETHYLASE3 (CMT3). The H3K9 demethylase INCREASED IN BONSAI METHYLATION 1 (IBM1) counteracts the detrimental consequences of KYP-CMT3 activity in transcribed genes. *IBM1* expression in *Arabidopsis* is uniquely regulated by methylation of the 7th intron, allowing it to monitor global H3K9me2 levels. We show the methylated intron is prevalent across flowering plants and its underlying sequence exhibits dynamic evolution. We also find extensive genetic and expression variations in *KYP*, *CMT3*, and *IBM1* across flowering plants. We identify *Arabidopsis* accessions resembling weak *ibm1* mutants and Brassicaceae species with reduced *IBM1* expression or deletions. Evolution towards reduced IBM1 activity in some flowering plants could explain the frequent natural occurrence of diminished or lost CMT3 activity and loss of gene body DNA methylation, as *cmt3* mutants in *A*. *thaliana* mitigate the deleterious effects of IBM1.

## Author summary

In flowering plants, the IBM1 histone demethylase plays a crucial role in regulating chromatin structure by removing H3K9me2, a modification associated with heterochromatin. This process involves a distinctive mechanism where the methylation of a repetitive sequence within an intron allows IBM1 to monitor and respond to global H3K9me2 levels. We discovered that this intron methylation sensor is widely observed across flowering plants, although the exact sequences involved show considerable variation. This suggests that while the mechanism for controlling IBM1 activity is conserved, it has adapted differently in various plant species. Additionally, our findings include the identification of *Arabidopsis thaliana* accessions that mimic weak *ibm1* mutants and several Brassicaceae species with diminished *IBM1* expression or deletions, which correlate with reduced CMT3 activity and gene body DNA methylation.

## Introduction

DNA methylation and histone H3 lysine 9 (H3K9) methylation are essential repressive chromatin modifications required for the formation of heterochromatin and the silencing of transposable elements (TEs), thereby playing a key role in maintaining genomic stability [1–5]. In plants, DNA methylation is observed in three different contexts: CG, CHG, and CHH (where H—A, C or T), each maintained by specific DNA methyltransferases. METHYLTRANSFERASE1 (MET1) is responsible for sustaining CG methylation through DNA replication [6], CHROMOMETHYLASE3 (CMT3) facilitates CHG methylation working in concert with the H3K9 methyltransferase KRYPTONITE (KYP) (also called SUVH4) [6–10], whereas CHH methylation is established either through the activities of CMT2 or the RNA-directed DNA methylation (RdDM) pathway [10,11]. All three contexts of DNA methylation are predominantly localized in heterochromatin and TE/repeat regions where CHG methylation (mCHG) is particularly important for reinforcing heterochromatin DNA methylation in conjunction with H3K9me2 [7,8,12–17]. This synergy is largely due to the unique characteristics of the enzymes CMT3 and KYP, as CMT3 preferentially binds to H3K9me2, and uses it as a guide to deposit mCHG and KYP, which recognizes pre-existing DNA methylation, adds H3K9me2 [8,15–17]. This interplay between CMT3 and KYP establishes a positive feedback mechanism, reinforcing the accumulation of both mCHG and H3K9me2 within heterochromatin regions [14,16].

The binding activity of KYP is not limited to mCHG, as KYP also engages with mCG prominently present in a specific group of genes classified as gene body methylated (gbM) [15,17–20]. These gbM genes typically include ‘housekeeping’ genes with moderate expression, characterized by extended gene lengths, lower substitution rates (dN/dS), a higher prevalence of CWG (W = A or T, cytosines preferred by CMT3), and fewer CG dinucleotides [21–26]. Despite the ongoing debate about the role of gbM in plants, one study suggests its role could be to suppress antisense transcripts within a subset of gbM regions [27]. One prevailing hypothesis is that CMT3 is important for the initial establishment of gbM, primarily through influencing CHG methylation [28–31]. This activity is thought to subsequently facilitate CG methylation in the gene body. Once established, CG methylation is maintained by MET1, ensuring the stability of methylation patterns across cell divisions and subsequent generations [6]. The observed natural absence of *CMT3* in some angiosperm species correlates with a loss of gbM and also highlights its role in a maintenance phase [28,31]. MET1 is the primary contributor to the maintenance of CG methylation within gene bodies, which is generally unaffected in Arabidopsis *cmt3* mutants [28]. However, genetic evidence supports its function in de novo mCG as well [32]. The presence of mCG within gbM genes likely facilitates KYP binding, recruiting the CMT3-KYP heterochromatin complex and exposing these genes to silencing machinery. However, the CMT3-KYP heterochromatin feedback loop in genic regions is disrupted by the histone lysine demethylase, INCREASED IN BONSAI METHYLATION1 (IBM1), which selectively demethylates H3K9me2 in genes, thus safeguarding them from silencing [33–36]. This protective role of IBM1 is underscored in *ibm1* mutants, which exhibit diverse phenotypic abnormalities and an accumulation of H3K9me2 and mCHG in approximately one-fifth of coding genes [33,34,36–38]. These affected genes in *ibm1* predominantly belong to the category of gbM genes [39], indicating a targeted recruitment of the CMT3/KYP complex to these specific loci. The dynamic interplay between IBM1 and CMT3/KYP is important for maintaining the equilibrium between euchromatin and heterochromatin, suggesting a co-evolutionary relationship [39]. In *Arabidopsis thaliana*, the seed fertility defect and meiotic abnormalities observed in *ibm1* is rescued by knocking out *CMT3*, indicating a functional interdependence [36,38]. Furthermore, the exclusive presence of both *IBM1* and *CMT3* in flowering plants supports the evolutionary connection between these two genes [30].

A unique aspect of *IBM1* is its dependency on DNA and H3K9 methylation within its large 7th intron for transcriptional and post-transcriptional regulation [40–42]. *IBM1* is a ubiquitously expressed gene and is known to produce two distinct mRNA isoforms in *A*. *thaliana*. *IBM1-L*, the longer isoform, encodes a functional protein with a catalytic JmjC histone demethylase domain, whereas its shorter counterpart, *IBM1-S*, is non-functional without the catalytic JmjC domain [40]. Notably, the expression of these isoforms is influenced by DNA methylation within the *IBM1* intron. In the case of *A*. *thaliana* Col-0, the 7th intron of *IBM1* contains DNA methylation, crucial for the expression of the functional *IBM1-L* isoform [40–42]. Full-length IBM1 has the capability to remove H3K9me2 in genic regions, suggesting that intron methylation in *IBM1* serves as a regulatory sensor of H3K9me2 by modulating the balance of its transcript isoforms [33,34,40]. Furthermore, our previous study revealed that certain natural *A*. *thaliana* accessions exhibit increased mCHG in genic regions, some of which also show decreased intron methylation in *IBM1*, along with an alteration in the *IBM1-S/IBM1-L* ratio compared to Col-0 [26]. This suggests a potential interaction between *IBM1* expression level and intron methylation level in *A*. *thaliana* accessions, and raises questions about the extent to which this intron methylation sensor mechanism is conserved among different *A*. *thaliana* accessions and across other flowering plant species in shaping the epigenome.

This study explores the association between intron DNA methylation of *IBM1* and its role in its own expression by surveying within and between species variation. *A*. *thaliana* accessions were identified that were reminiscent of weak *A*. *thaliana ibm1* mutants, as they possessed ectopic mCHG in a subset of genes. Furthermore, a comparative analysis of *IBM1* orthologs across 34 angiosperm species demonstrated the presence of intronic DNA methylation within its 7th intron, indicating the evolutionary conservation of the H3K9me2 sensor in flowering plants. However, the sequence underlying the methylated intron was highly variable between species suggesting this heterochromatin sensing activity exhibits significant evolutionary divergence. Moreover, our investigation into multiple Brassicaceae species suggests the coevolution of *IBM1* and *CMT3* within this family and likely all flowering plants. This is particularly evident in Brassicaceae species that lack gbM, such as *Eutrema salsugineum* and *Thlaspi arvense*, as we observed a correlation between low or absent *CMT3* expression and reduced *IBM1* expression. This association was further supported by DNA methylome data from other Brassicaceae species that have reduced/absent *IBM1* and/or *CMT3* function as well as gene body DNA methylation. Collectively, our study shows that *IBM1*, its intronic heterochromatin sensor and *CMT3* are dynamically evolving and that this shapes the genic methylation landscape in plants.

## Results

### Reduced intronic methylation of *IBM1* is associated with ectopic genic hypermethylation in natural *A*. *thaliana* accessions

IBM1 is a histone demethylase that removes H3K9me2 from transcribed regions in *A*. *thaliana* [33,35,36]. The functional loss of *IBM1* results in an accumulation of H3K9me2 and non-CG methylation (mCHG) in a subset of gene bodies [34,36,39,40]. The 7th intron of *IBM1* is able to sense genome-wide levels of H3K9me2, which affects *IBM1* transcription and H3K9 demethylase activity to maintain H3K9me2 levels genome wide [40]. Mutants deficient in DNA methylation, such as *met1* and *cmt3*, exhibit a decrease in the transcription of full-length *IBM1* transcripts along with a loss or reduction of DNA methylation in the *IBM1* intron region [40]. In *met1*, decrease of *IBM1* expression induces the ectopic gain of mCHG and H3K9me2 in multiple genes [40], similar to how it occurs in *ibm1* [33–36,39]. Intriguingly, our previous research has identified three natural *A*. *thaliana* accessions exhibiting a weak *ibm1*-like phenotype, characterized by ectopic mCHG-gain in a subset of genes [39]. These accessions also showed a decrease in the DNA methylation level within *IBM1’s* 7th intron, suggesting that the heterochromatin sensor activity of *IBM1* is potentially evolving [39].

**Fig 1:**
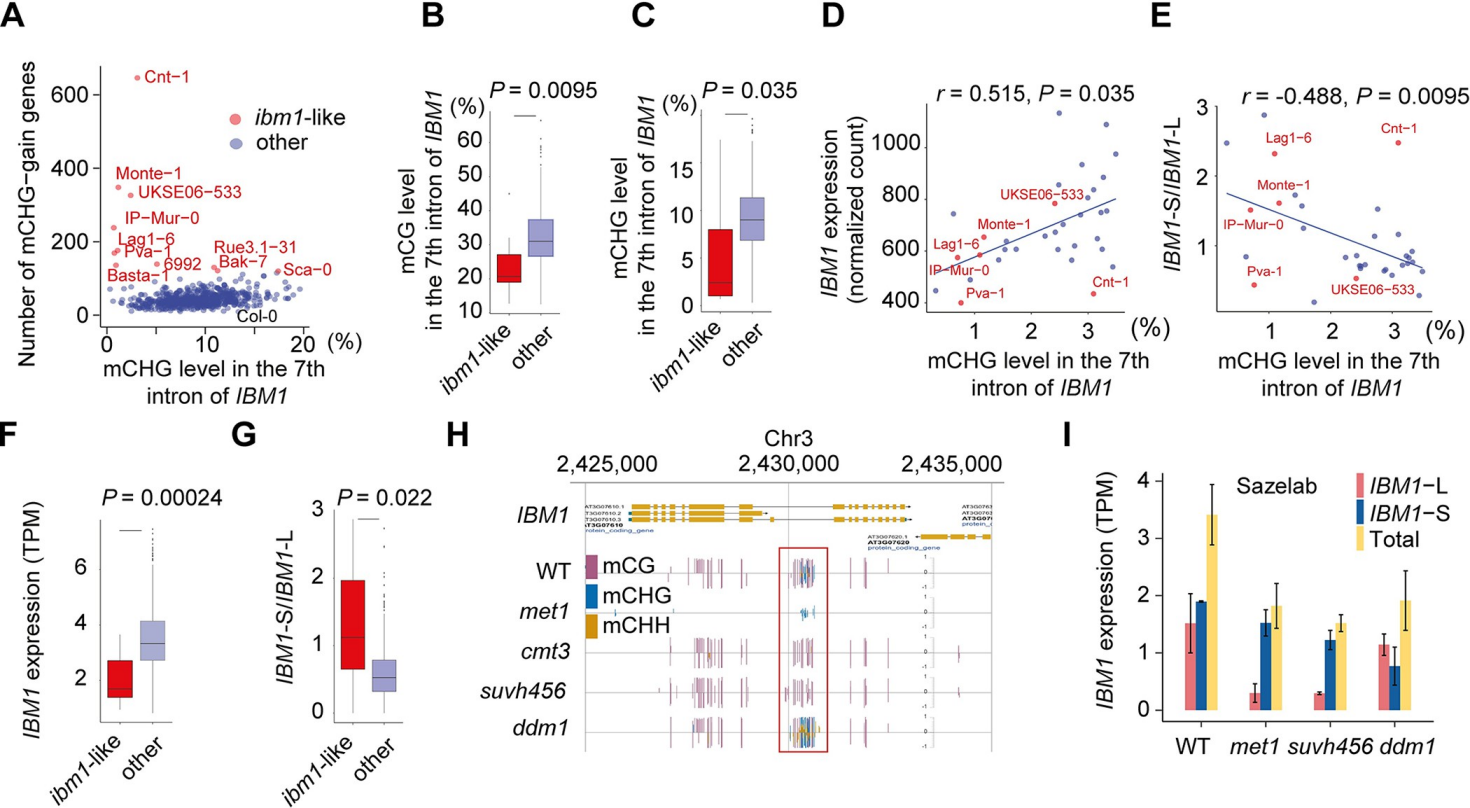
Natural variation in intron DNA methylation of *IBM1* within *A*. *thaliana*. **(A)** The scatter plot shows the mCHG levels in *IBM1*’s long 7th intron against the number of genes with ectopic non-CG methylation in each accession. Accessions with a significant number (n>120) of ectopic methylated genes are labeled by name and marked with red dots, whereas other accessions are represented with blue dots. The box plot displays the distribution of **(B)** mCG and **(C)** mCHG levels in the long intron of *IBM1*, comparing the top 11 accessions (labeled in A) with the highest number of ectopic methylated genes against all other accessions. (**D)** The scatter plot shows the relationship between mCHG levels in the long intron of *IBM1* and *IBM1* gene expression for accessions with low *IBM1* long intron mCHG levels (< = 0.035). The dots are colored following the same scheme as in (A). **(E)** Similar to D, this scatter plot presents the mCHG level in the long intron of *IBM1* plotted against the ratio of short to long isoform of *IBM1*. **(F)** The box plot shows the distribution of *IBM1* gene expression by comparing accessions that rank in the top 11 for the highest number of ectopic methylated genes with all remaining accessions. **(G)** Similar to F, the box plot shows the distribution of the ratio of short to long isoform of *IBM1* between the two groups. **(H)** The browser shows the intron methylation of *IBM1* in Col-0 WT, *met1*, *cmt3*, *suvh456* and *ddm1* mutants. **(I)** The bar plot shows the gene expression levels of the long and short isoforms of *IBM1*, as well as their combined expression, in *ddm1*, *met1*, *suvh456* mutants, and Col-0 WT. The isoform expressions were quantified using RNA-seq data sourced from the Saze lab [47].

To further explore the relationship between intron methylation of *IBM1* and ectopic mCHG in gene bodies, we used RNA-seq isoform quantification using transcriptomes from the 1,001 genomes project (n = 635 with both RNA-seq and DNA methylation data) [16,43–45]. Our analysis revealed that eleven *A*. *thaliana* accessions possess ectopic mCHG in at least 120 genes (Fig 1A, labeled top eleven by name), thereby categorizing them as *ibm1*-like accessions. Notably, these *ibm1*-like accessions tend to show lower intron methylation in both CG and CHG contexts compared to the rest of the population (Fig 1B and 1C). The observed decrease in intron methylation is not due to genetic variation among the accessions, as the DNA methylation mapping coverage of the heterochromatin sensing intron was comparable across the accessions (S1A–S1D Fig and S1 Table). Furthermore, in accessions exhibiting low mCHG levels within the *IBM1* intron, a positive correlation (p<0.035) was observed between intron DNA methylation levels and *IBM1* gene expression (Figs 1D and S1E–S1G). Conversely, there was a negative correlation with the proportion of short isoform *IBM1* transcripts (Figs 1E and S1H–S1K). Consistent with this observation, the *ibm1*-like accessions tend to exhibit reduced expression of *IBM1* and an increased proportion of the *IBM1-S/IBM1-L* transcript isoforms compared to other accessions (Fig 1F and 1G).

This diminished IBM1 activity likely contributed to the onset of ectopic genic methylation in these accessions. The reduced expression of functional *IBM1* in these accessions raises a question regarding the plant’s potential regulatory response, specifically whether there is a compensatory downregulation of *CMT3* expression to mitigate the effects of imbalanced genic methylation. However, our investigations reveal that *CMT3* expression levels did not show significant alterations in the accessions exhibiting an *ibm1*-like molecular phenotype (S1L Fig). Collectively, our findings indicate a robust association between the DNA methylation status of the *IBM1* intron, the ratio of the short and long transcript isoforms and the gene expression levels of *IBM1*. This relationship appears to be a significant factor influencing the widespread acquisition of mCHG in the gene bodies in a subset of *A*. *thaliana* accessions.

To validate the accuracy of RNA-seq based isoform quantification, we conducted a detailed analysis of *IBM1* isoform expression across various DNA methylation mutant lines using different data sources, including *met1*, *suvh456*, *cmt3*, *ibm2*, *ddm1*, and *drd1* (S2 Table) [11,41,46–48]. MET1 and CMT3 are responsible for maintaining CG and CHG methylation, respectively [6,7]. The SUVH4 (also called KYP), SUVH5 and SUVH6 proteins are involved in the H3K9me2 deposition, crucial for transposable element silencing and heterochromatin formation [13,17,41,42,49]. DDM1, a chromatin remodeler, is essential for maintaining heterochromatin across the genome [11]. DRD1 is also a chromatin remodeler which regulates RNA-directed DNA methylation pathway, which is required for silencing transposable elements through methylation [11,50]. Lastly, IBM2 plays an important role in ensuring RNA polymerase II processing at loci carrying heterochromatic transposable elements including *IBM1* locus [41,42]. In Arabidopsis, the interaction between an IBM2/EDM2/AIPP1 complex and the heterochromatic repeat in the *IBM1*’s intron is essential for regulating *IBM1* expression [51–53]. IBM2, an RNA-binding protein with a BAH domain, likely mediates chromatin association within the heterochromatin regions of the IBM1 intron, whereas EDM2 recognizes H3K9me2 within the intron [41,42,52]. AIPP1 serves as a bridge between IBM2 and EDM2, helping to form a protein complex that promotes full-length expression of the *IBM1* transcript [53]. This system of interactions ensures precise control of *IBM1* expression. The mutants showing a reduction in *IBM1* intron methylation, such as *suvh456*, *cmt3*, and *met1*, demonstrated a decrease in full-length *IBM1-L* expression level (Figs 1H,1I and S2A–S2C). In *ibm2*, where the gene encoding the enzyme crucial for *IBM1’s* proper transcription is affected [41,42], also exhibited significant reduction of the *IBM1-L* transcript (S2D Fig). Conversely, in *ddm1* and *drd1* where the intronic methylation of *IBM1* is unaltered, the transcription of the full-length transcripts remains unaffected (Figs 1I and S2C), indicating a direct correlation between intron DNA methylation status and the expression of functional *IBM1-L* isoform [40].

To assess the prevalence of the role of intron methylation in transcription, we analyzed all *A*. *thaliana* Col-0 genes with long introns (> 1kb). A total of 109 out of the 705 genes identified exhibit non-CG methylation within their introns (S3 Table). We observed that Col-0 genes with mCHG in the intron (n = 109) generally show less expression compared to those without mCHG (S2E Fig). However, for most Col-0 genes with mCHG in the introns, a reduction in methylation—ascertained by comparing *suvh456* mutants with wild-type plants—does not influence the production of full-length transcripts (S4 Table). The notable exceptions to this are *IBM1* and *PPD7 (AT3G05410)*, a component of the thylakoid lumen proteome essential for the photosystem II oxygen-evolving complex in chloroplasts (S2F and S2G Fig). This association was previously established in *ibm2* mutants [53]. Yet, in natural accessions, the pronounced effect of *PPD7’s* intron methylation reduction on *PPD7* transcription observed in mutants does not persist (S2I Fig). For instance, *PPD7’s* intron methylation is completely absent in the Cnt-1 accession, which surprisingly does not impact the generation of full-length transcript of *PPD7*. Intriguingly, minimal read mapping occurs within the *PPD7’s* intron region in Cnt-1, a long region (5.7 kb) with a non-LTR retrotransposon in the Col-0 accession (S2H and S2I Fig). This suggests a possible deletion of the entire region in Cnt-1. Although some accessions exhibit low read coverage, the majority demonstrate adequate coverage; yet, even with low mCHG levels, no substantial effect on the transcription process is evident in most accessions (S2I and S2J Fig). This indicates that factors beyond total intron deletion, such as TE sequences within these large introns, also may contribute to variations in transcriptional responses. This is supported by a recent study showing that intragenic transposable elements can modulate transcription initiation, termination and splicing [54].

### The ectopic mCHG in genes in Cnt-1 is reduced by *IBM1* overexpression

**Fig 2:**
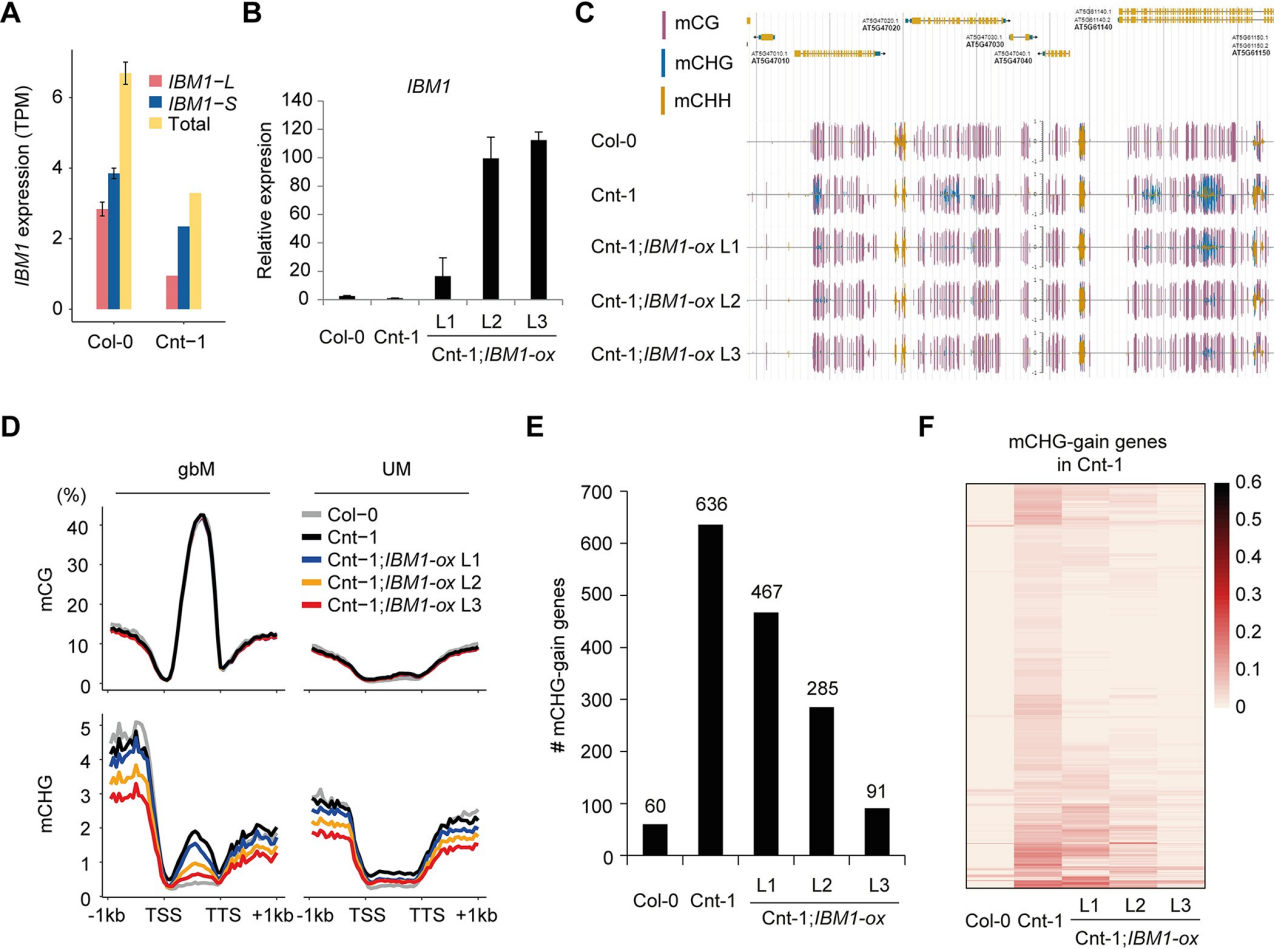
The ectopic mCHG in genes in Cnt-1 is reduced by *IBM1* overexpression. **(A)** Expression level of *IBM1-L* and *IBM1-S* in Col-0 and Cnt-1. **(B)** Expression level of *IBM1* in each transgenic plant. Primers were designed to target the conserved region of *IBM1* between Col-0 and Cnt-1. RT-qPCR was performed to estimate the expression level of *IBM1* in these plants. **(C)** A genome browser view shows that ectopic mCHG in genes in Cnt-1 is reduced by introducing the *IBM1* transgene. **(D)** Metaplots show the average changes to mCHG over gbM and UM genes in *IBM1* transgenic lines of Cnt-1. **(E)** The number of mCHG-gain genes is reduced in *IBM1* transgenic lines compared to Cnt-1. **(F)** Heatmaps show reduction of mCHG on genes in *IBM1* transgenic lines of Cnt-1. The 636 mCHG-gain genes from (E) are shown.

The *A*. *thaliana* Cnt-1 accession stands out as an exceptional case, exhibiting the largest number of ectopic mCHG-gain genes among the natural accessions along with lower levels of intron methylation (Fig 1A). As this phenotype resembles that of a weak *ibm1* mutant, we explored isoform quantification analysis of Cnt-1 to estimate *IBM1’s* expression level. Notably, Cnt-1 displayed a substantially lower expression level of the functional *IBM1-L* isoform in comparison to the Col-0 reference accession, with only a marginal reduction in the expression of the *IBM1-S* isoform (Fig 2A). This result suggests that decreased expression of *IBM1-L* in Cnt-1 leads to an elevation in mCHG within these genes. Moreover, TE methylation in Cnt-1 is relatively high among all accessions (S1M Fig and S1 Table). This combination of subdued IBM1 activity and vigorous CMT-KYP methylation may account for the high number of mCHG-gain genes in Cnt-1 and supports that this heterochromatin sensing mechanism is dynamic in populations to shape the epigenome.

Next, we examined the effect of restoring *IBM1* expression in the Cnt-1 accession. The *UBQ10* cis-regulatory sequences were used to express the Col-0 *IBM1* coding sequence in Cnt-1, resulting in the generation of three independent transgenic lines. These lines exhibited a significant increase in *IBM1* expression, ranging from 20-100-fold higher than the control (Fig 2B). All three of the *IBM1*-ox lines displayed a decrease in mCHG in a subset of gene bodies compared to the Cnt-1 control (Fig 2C and 2D), indicating that overexpression of *IBM1* reduces ectopic mCHG in gene bodies presumably by decreasing H3K9me2, although this was not tested. In Arabidopsis, H3K9me2 and mCHG form a self-reinforced loop, thus, measuring mCHG levels can indirectly reflect H3K9me2 levels [8,16,17]. This suggests that the observed reduction in mCHG are likely indicative of decreased H3K9me2 levels. This reduction was further supported by a decrease in the number of mCHG-gain genes when compared to the Cnt-1 control (Fig 2E). A heatmap analysis of mCHG-gain genes from Cnt-1 revealed a substantial decrease in mCHG levels in the *IBM1*-ox lines (Fig 2F). Despite the substantial increase in expression levels (Fig 2B), complete mCHG reduction was not achieved (Fig 2C–2F), which could be attributed to promoter choice. Although we used the *UBQ10* promoter for its strong and constitutive expression, this may not fully replicate the native expression pattern of *IBM1*, particularly in tissue-specific contexts where IBM1 function is crucial, such as during meiosis [38]. This discrepancy could explain the partial reduction in mCHG levels observed, suggesting that the expression driven by *UBQ10* does not fully mimic the native promoter’s regulatory effects. Considering the conservation of *IBM1* coding sequences between Col-0 and Cnt-1 (99.61% sequence identity), these findings collectively indicate that increasing *IBM1* expression in Cnt-1 significantly reduces the aberrant mCHG accumulation in the gbM genes. We extended the analysis of methylation beyond gene regions to include TEs and TE genes (S3A Fig). When comparing the methylation profiles of the Col-0 and Cnt-1 lines, both generally exhibited similar methylation levels. However, the *IBM1-ox* lines displayed methylation patterns similar to Cnt-1 for mCG, while mCHG and mCHH levels were slightly reduced. Furthermore, we assessed global DNA methylation levels (S3B Fig), revealing that, aside from minor differences in mCG levels compared to the Cnt-1 control, there were notable reductions in mCHG and mCHH levels across the pericentromeric regions of the genome. This suggests that *IBM1* overexpression has a broad impact on methylation, affecting regions well beyond gene bodies.

### Methylation of the intronic heterochromatin sensor is a common feature in *IBM1* orthologs across flowering plants

We noted a heightened mutation frequency within the methylated intron of *IBM1* compared to the unmethylated non-coding regions and the coding sequences of *IBM1* across *A*. *thaliana* populations as well as at the species level (S4A and S4B Fig and S5 Table). In addition, methylated long introns in *A*. *thaliana* (> 1kb) exhibit a higher mutation frequency, suggesting the inherent mutagenicity of DNA methylation in *A*. *thaliana* populations. (S4C Fig). Given these observations, further exploration into the variability of *IBM1* intronic DNA methylation and the conservation of its regulatory role across different species presents an intriguing line of inquiry. To investigate the patterns of intronic DNA methylation of *IBM1* across angiosperm species, we first identified the orthologous genes of *IBM1* in various plant species. This was achieved through the construction of a Maximum Likelihood (ML) gene tree, using 1,516 JmjC family homologous genes derived from 597 different species (see Methods and S6 Table). The orthologs of *IBM1* were defined as those genes that clustered within the same clade as *A*. *thaliana’s IBM1*, specifically clade 5 (S4D–S4F Fig).

**Fig 3:**
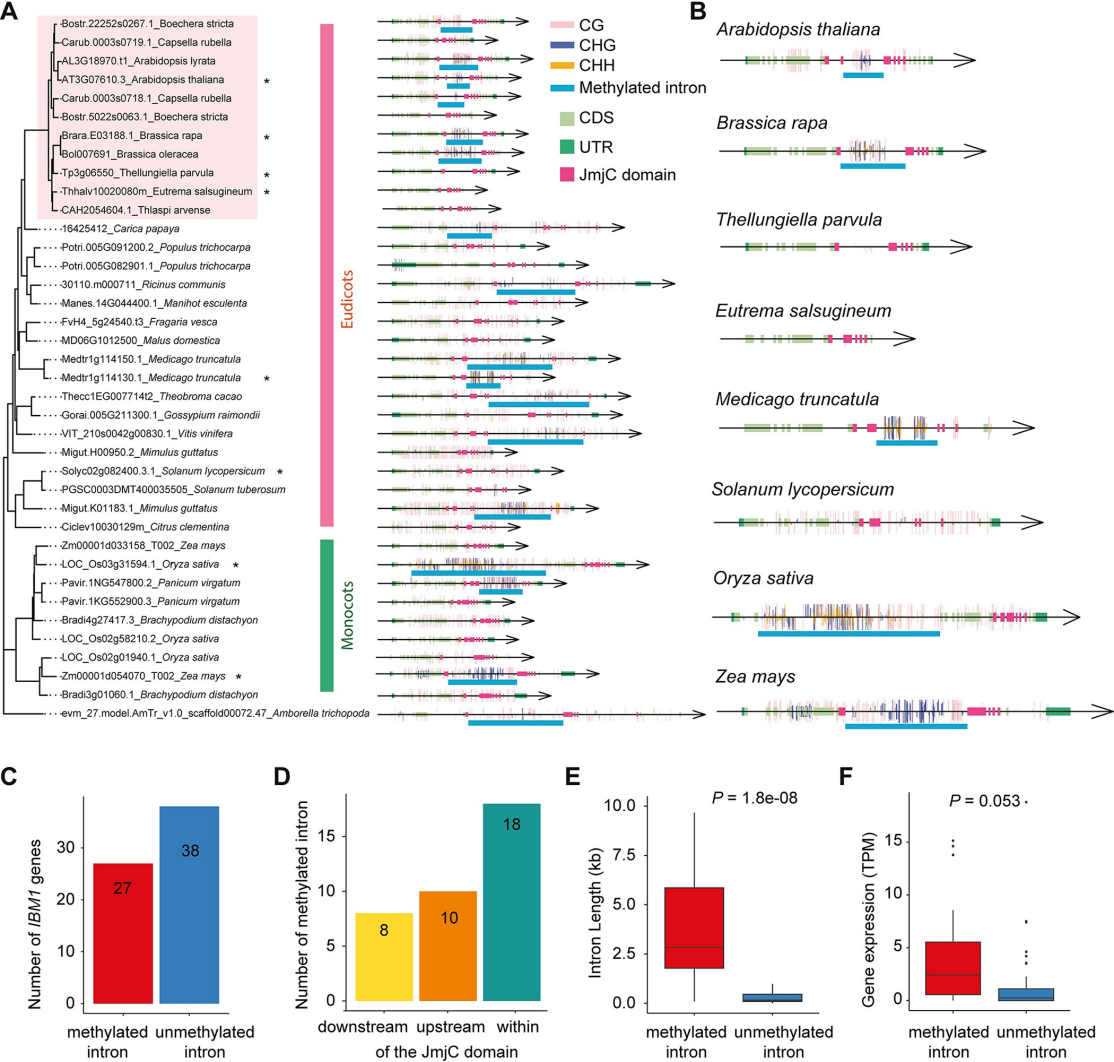
Intron methylation is widely present in *IBM1* homologous genes of angiosperms. **(A)** The maximum likelihood gene tree displayed here represents the *IBM1* orthologs from 34 angiosperm plants that have DNA methylome data. Due to space constraints, this is a truncated version of the tree; the complete version is available in S5 Fig. The left panel presents the gene tree, while the right panel depicts the corresponding gene structures. UTRs, CDS, and the JmjC domain are highlighted in different colors, as indicated in the legend, with introns represented by thin black lines. The locations of methylated sites on the gene sequences are marked with vertical bars, with distinct colors denoting the different methylation contexts: CG, CHG, and CHH. **(B)** Zoomed-in version of (A). A few species were enlarged to highlight the intron methylation. **(C)** The bar plot shows the number of genes out of the 65 *IBM1* orthologs investigated that are either with or without mCHG in their introns. To assess the significance of mCHG levels within these introns, a binomial test was applied. **(D)** The bar plot displays the count of introns with mCHG relative to their position within, before, or after the JmjC domain. It should be noted that a single *IBM1* ortholog may contain multiple methylated introns. **(E)** The boxplot shows the distribution of intron lengths between *IBM1* gene copies with methylated introns and those with unmethylated introns. **(F)** The boxplot shows the distribution of expression levels between *IBM1* gene copies with methylated introns and those with unmethylated introns.

We analyzed the DNA methylation patterns on *IBM1* orthologous genes in 34 plant species for which DNA methylome data were available (S7 Table) [19]. This set includes *A*. *trichopoda*, the most basal species in our study, which was used as an outgroup. Our investigation focused on identifying introns within these *IBM1* orthologs that exhibit significantly higher levels of mCHG methylation compared to the species-specific background genic methylation levels (S8 Table). Our findings reveal that intronic CHG methylation is prevalent in *IBM1* orthologous genes across both eudicots and monocots (Figs 3A,3B and S5A–S5C). Among the 65 *IBM1* orthologs analyzed, which include species with multiple gene copies, 27 *IBM1* orthologs were significantly enriched for mCHG within their introns (Fig 3C).

Within the Brassicaceae family, intronic DNA methylation is present in the *IBM1* orthologs in six out of nine species, encompassing all species from the *A*. *thaliana* subclade (Fig 3A). The intronic methylation observed in these *IBM1* orthologs is predominantly found within the JmjC domain, a pattern that is consistent with what has been observed in *A*. *thaliana* (Fig 3B). A varied pattern emerges in the *E*. *salsugineum* subclade: *B*. *rapa* and *B*. *oleracea* exhibit significant mCHG enrichment in the long intron region within the JmjC domain, whereas *T*. *parvula* and the species with shorter introns, *E*. *salsugineum* and *T*. *arvense*, show a loss of mCHG (Fig 3A and 3B). This is noteworthy, as the species with no or reduced DNA methylation in the *IBM1* intron region containing the heterochromatin sensor are the species that have lost gbM [28,55]. For species outside the Brassicaceae family, the patterns of intronic methylation in *IBM1* orthologs are more variable and not strictly confined to the JmjC domain. DNA methylation is observed in regions both upstream and downstream of the JmjC domain (Figs 3A, 3D and S5B), and this variability in DNA methylation location does not appear to be conserved in species from the same family. For instance, in the Fabaceae family, the *IBM1* orthologs in *L*. *japonicus* and *G*. *max* exhibit intronic methylation after the JmjC domain, whereas *M*. *truncatula* from the same family shows DNA methylation within the JmjC domain (S5C Fig). A similar pattern is noted in the Poaceae family: an *IBM1* ortholog in *P*. *virgatum* has intronic DNA methylation within the JmjC domain, whereas in *O*. *sativa*, it is located before the JmjC domain. The mCHG introns are typically more enriched in longer introns (Fig 3E). For instance, a gene copy in *M*. *esculenta*, with three successive large introns (7.3kb, 3.6kb, 9.6kb), and another in *T*. *cacao*, with a 27.2 kb intron, both exhibit significant DNA methylation enrichment within these intron regions (S5A and S5B Fig). Interestingly, the *IBM1* orthologs with mCHG introns also exhibit higher expression levels. (Fig 3F). These data support that many of these species likely use a similar mechanism to *A*. *thaliana* to sense heterochromatin content given the persistence of intron methylation within the JmjC domain of IBM1.

### The DNA sequence underlying the heterochromatin sensor of *IBM1* is evolving

**Fig 4:**
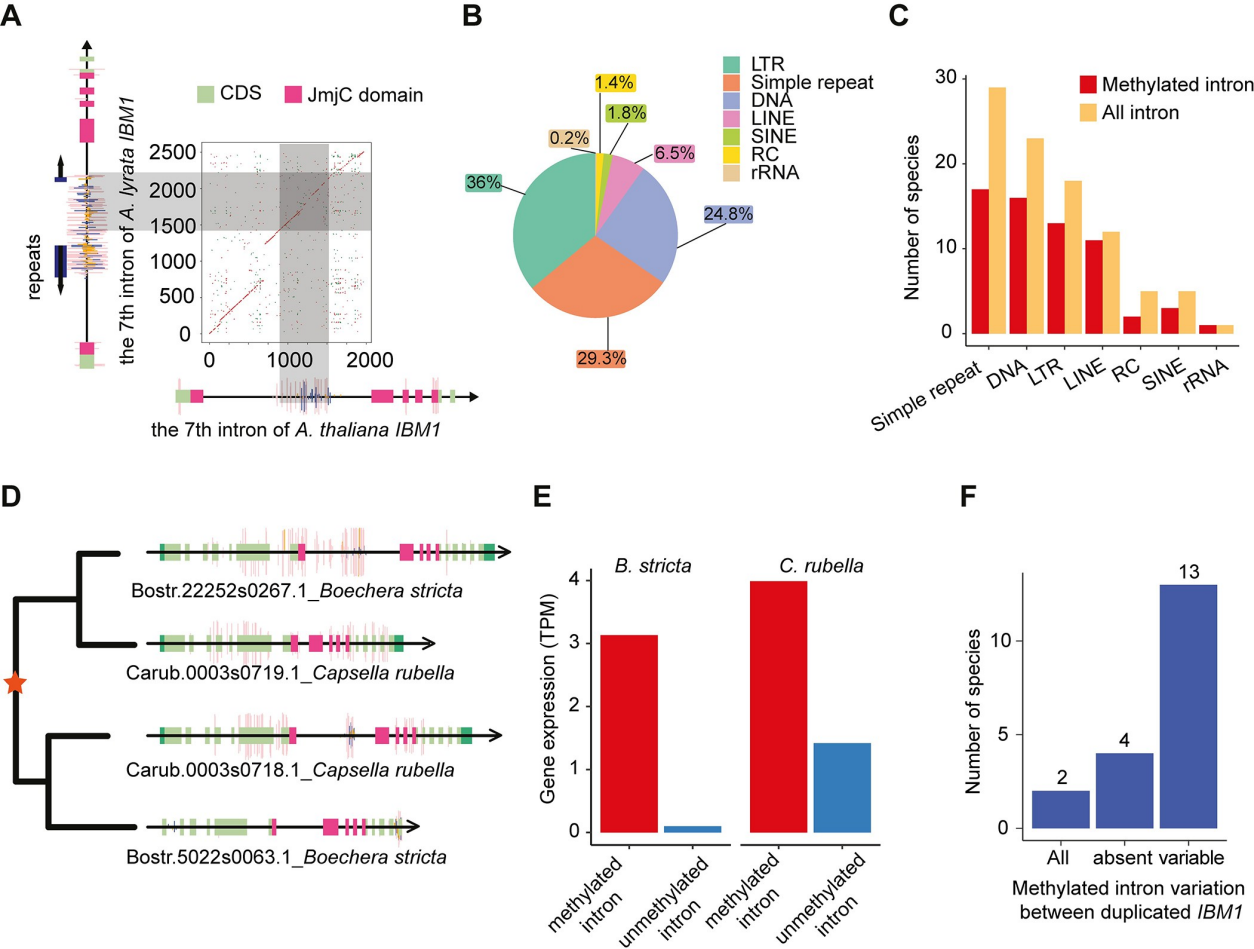
The sequence underlying the methylated intron of *IBM1* is highly divergent between species. **(A)** The plot illustrates the sequence comparison between *A*. *lyrata* and *A*. *thaliana*, which reveals an insertion of approximately 500 bp in the intron of *A*. *lyrata*. This inserted fragment comprises repeat sequences that are methylated. **(B)** The pie chart shows the proportion of DNA repeat types found in the introns covered by mCHG across the 65 *IBM1* orthologs. **(C)** The bar plot illustrates the distribution of primary DNA repeat types across the introns of *IBM1* orthologs in 34 species. **(D)** Gene structure diagrams show the different intron methylation patterns of duplicated *IBM1* genes in *B*. *stricta* and *C*. *rubella*. **(E)** The bar plot displays the expression levels of duplicated IBM1 genes, categorized by the presence or absence of intronic methylation in *B*. *stricta* and *C*. *rubella*, respectively. **(F)** The bar plot displays the distribution of intronic methylation variation among 19 species that have duplicated *IBM1* gene copies. The categories are defined as follows: ’All’ indicates that every *IBM1* gene copy in a species has a methylated intron, ‘absent’ signifies that no copies of the *IBM1* genes in a species have methylated introns, and ’variable’ denotes species where the methylation status differs among the *IBM1* gene copies.

In *A*. *thaliana*, DNA methylation in the 7th intron is present within a 150-bp repeat sequence that shows high similarity to *YCF1*, a gene encoded by the chloroplast genome [41]. Organellar sequences are often silenced by DNA methylation upon integration into the nuclear genome. In the *IBM1* ortholog from *A*. *lyrata*, the DNA methylated region in the 7th intron is larger than that in *A*. *thaliana*, as it arises from two insertions of simple repeat elements approximately 200 bp upstream and 50 bp downstream of the sequence aligned with the DNA methylated intron of *A*. *thaliana* (Fig 4A). Intriguingly, this *YCF1*-like fragment is identified in *A*. *lyrata*, but not other Brassicaceae species, suggesting significant evolutionary divergence (S9 Table).

It is likely the heterochromatin sensing ability of *IBM1* is conserved throughout many flowering plants given the presence of mCHG in introns within the JmjC domain of many species (Fig 3A and 3D). However, the sequence underlying the methylated introns is not conserved within species (Fig 4B, 4C and S9 Table). We found numerous alignments of simple and transposon repeats showing that sequence evolution within the intronic heterochromatin sensing region of *IBM1* is quite dynamic between species (Fig 4B and 4C). For species that have a candidate heterochromatin sensor in an *IBM1* intron, the actual sequence that is methylated isn’t as important as the methylation event itself. The fact that the sequence is continually changing between species, but remains methylated in many flowering plants suggests that there could be constant gains and loss of *IBM’s* ability to sense H3K9me2 levels over evolutionary time.

In fact, DNA methylation patterns in the intronic region of *IBM1* orthologs not only varied among different species, but also among duplicated gene copies within the same species. For instance, a tandem duplication event happened just before the speciation of *B*. *stricta* and *C*. *rubella*, resulting in each species possessing two *IBM1* gene copies, and DNA methylation and gene expression profiles also varied between the two gene copies (Fig 4D and 4E). Approximately 20% of species in this clade possess duplicated *IBM1* genes (S4G Fig) and out of the 34 species for which there is DNA methylation data, 19 species contain duplicated *IBM1* genes, and over half exhibit DNA methylation pattern divergence between the duplicates (Fig 4F). This indicates that upon duplication the heterochromatin sensor activity is not retained in duplicate copies.

### Gene body DNA methylation is frequently lost in Brassicaceae species due to reduction of CMT3 or IBM1 activity

**Fig 5:**
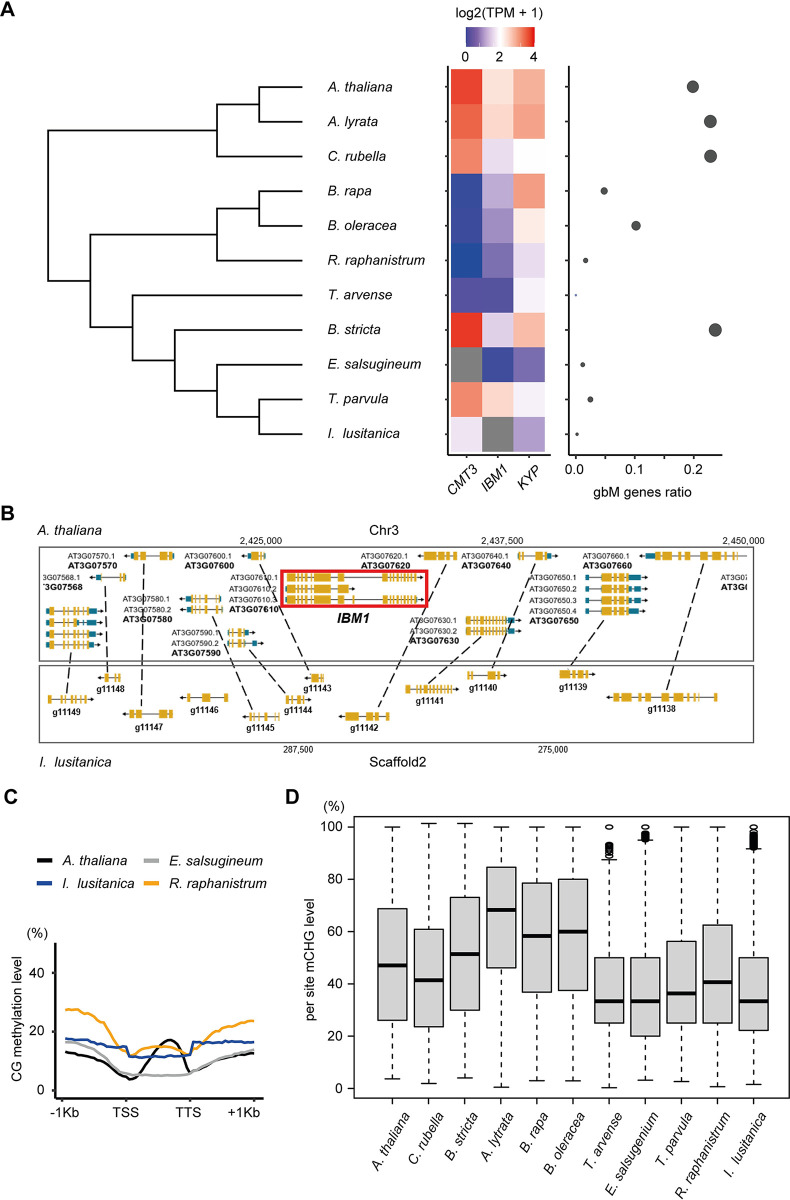
Frequent expression and genetic variation of *IBM1* and *CMT3* in Brassicaceae. **(A)** The heatmap on the left panel displays the expression levels of *CMT3*, *IBM1* and *KYP* in Brassicaceae species. On the right panel, the scatter plot shows the gbM gene ratio for each species, which is calculated as the number of gbM genes divided by the total number of genes where DNA methylation could be measured in that species. The size of the points in the scatter plot is proportional to the gbM gene ratio. **(B)** A genome browser view of a region of synteny between *I*. *lusitanica* and *A*. *thaliana* depicting the absence of *IBM1*. **(C)** The mCG metaplots display the methylation patterns across all genes in newly analyzed Brassicaceae species *I*. *lusitanica* and *R*. *raphanistrum*. **(D)** Box plots showing per-site mCHG level across the genome, which reflects the enzyme activity of CMT3.

Previous studies have shown that loss of *CMT3* or reduced CMT3 activity is associated with a loss or a reduction of gbM [28,31]. Importantly, the loss of *CMT3* has occurred multiple independent times suggesting that this is an evolving process [28,31]. One possible reason for the loss of *CMT3* or a reduction in its activity could be due to reduced function of IBM1. Loss of IBM1 activity in *A*. *thaliana* leads to reduced expression of gbM genes and eventually lethality [36]. However, this is rescued by reduced activity or a complete loss of CMT3 [36,38]. To explore this possibility further, we analyzed transcriptomes and DNA methylome data from various Brassicaceae species, specifically focusing on *CMT3*, *IBM1*, and *KYP* expression and the prevalence of gbM (Fig 5A). In *E*. *salsugineum*, a noticeable decrease in *IBM1* expression accompanies the loss of *CMT3*, coinciding with a substantial reduction in gbM. A similar pattern emerges in *T*. *arvense*, closely related to *E*. *salsugineum*. Although *T*. *arvense* retains *CMT3*, both *IBM1* and *CMT3* exhibit low expression levels, correlating with an absence of gbM. This trend persists within the Brassicaceae subclade; species like *B*. *rapa* and *B*. *oleracea*, showing diminished *CMT3* and *IBM1* expression, also display a marked decrease in gbM genes (Fig 5A and S10 Table). This is consistent with our previous results showing *CMT3* in some Brassicaceae is under relaxed selective constraints [30].

By mining publicly available reference Brassicaceae genomes, we identified two additional species (*Raphanus raphanistrum* and *Isatis lusitanica*) with potential loss or truncations of *IBM1* or *CMT3* (S6A Fig). However, the quality of these reference genomes was quite variable, requiring additional experiments to validate the loss or truncations of *IBM1* or *CMT3*. We used transcriptome assembly as well as PCR of genomic DNA using universal primers to extract full-length *IBM1* or *CMT3* loci (S11 Table). We were unable to identify *IBM1* in *I*. *lusitanica* suggesting it might be lost in this species. There is clear evidence that it is deleted within a region of synteny with *A*. *thaliana* (Fig 5B). We successfully identified *CMT3* sequences across both species, although *I*. *lusitanica* exhibited a small N-terminal deletion in *CMT3* (S6B Fig and S12 Table). Next, we estimated the expression level of *IBM1*, *CMT3* and *KYP* across these species and evaluated the presence of gbM genes (Fig 5A). The number of potential gbM detected in *I*. *lusitanica* and *R*. *raphanistrum* was as low as *E*. *salsugineum* (Fig 5A and 5B) indicating it has been lost in these species. In *R*. *raphanistrum*, *CMT3* expression was absent, coupled with a significant reduction in *IBM1* expression. In *I*. *lusitanica*, in which we couldn’t identify *IBM1*, there were relatively lower *CMT3* expression levels compared to Brassicaceae species with high numbers of gbM genes (Fig 5A). We also assessed the expression levels of *CMT1* and *CMT2*, genes orthologous to *CMT3*, in species exhibiting reduced or absent *IBM1* expression, finding no significant changes in their expression levels (S7 Fig).

When measuring gene body mCG, *A*. *thaliana* exhibited a typical bell-shaped gbM pattern (Fig 5C). However, both *I*. *lusitanica* and *R*. *raphanistrum* lacked this pattern, similar to *E*. *salsugineum*, which correlates with a substantial reduction of gbM (Fig 5A and 5C). Notably, the basal mCG levels in *I*. *lusitanica* and *R*. *raphanistrum* were slightly higher than in *E*. *salsugineum* (Fig 5C). This could be the result of higher methylation levels in TE-like methylated genes, which increases basal mCG levels. This is further supported by the fact that mCHG levels in *I*. *lusitanica* and *R*. *raphanistrum* were also slightly higher than in *E*. *salsugineum* (S8A Fig). We also analyzed the methylation levels in TEs. Our analyses reveal that *R*. *raphanistrum* exhibits higher methylation levels of both CG and CHG in TEs compared to *A*. *thaliana*, while *I*. *lusitanica* shows higher CG but lower CHG methylation levels than *A*. *thaliana* (S8B Fig). These differences in TE methylation patterns might contribute to the observed variations in gene body methylation and overall methylation profiles among these species. We hypothesized that even though some of the Brassicaceae species have *CMT3*, its expression or enzymatic activity might be reduced or absent, consistent with the reduction of gbM (Fig 5A). We tested this by measuring mCHG levels and mCHG symmetry, both of which should be present if CMT3 activity is functional. Genome-wide per-site mCHG levels were measured across species, revealing *R*. *raphanistrum* and *I*. *lusitanica* with low mCHG levels, similar to gbM-absent species such as *E*. *salsugineum* (Fig 5D). In Arabidopsis, mCCG is underrepresented compared to other CHG sites, as the CCG methylation depends on MET1 as well as CMT3 [56]. We have conducted additional per site methylation analysis on CCG sites and confirmed that they show low mCCG level in Arabidopsis as expected (S8C Fig). We also assessed the methylation symmetry at CWG sites (W = A or T) preferentially targeted by CMT3. Furthermore, *R*. *raphanistrum* and *I*. *lusitanica* exhibited a predominantly asymmetric mCWG pattern, aligning with the pattern observed in *E*. *salsugineum*, which lacks *CMT3* (S9 Fig).

## Discussion

The discovery of heterochromatin sensing activity associated with *IBM1* in *A*. *thaliana* supports that mechanisms have evolved to buffer heterochromatin abundance in flowering plant genomes [26,40]. This discovery parallels the DNA methylation sensing activity in *A*. *thaliana* associated with the DNA demethylase *ROS1* [57,58]. Epigenome homeostasis is an emerging property associated with chromatin regulation and genome evolution [59,60]. The existence of these epigenome sensors suggests that mechanisms are in place to cope with wholescale changes to H3K9me2 or DNA methylation patterns in plants that could occur due to epigenome shock, whole genome duplication and/or hybridization events among other possibilities.

This study explored the evolution of heterochromatin sensor activity within an intron that disrupts IBM1 activity if it is not properly spliced in *A*. *thaliana*. We identified both within and between species variation of IBM1 activity. Some *A*. *thaliana* accessions were identified that had ectopic mCHG in gene bodies, which was rescued by ectopic expression of *IBM1*. Variation in the related histone lysine nine demethylase *JMJ26* was recently discovered in a genome-wide association study for mCHG variation in transposons [61]. This provides precedent for natural variation of histone demethylase activity in shaping plant epigenomes. Recent findings emphasize the developmental relevance of *IBM1* regulation, showing differential IBM1 activity due to tissue-specific methylation patterns [62]. For example, research indicates that in the endosperm, reduced methylation within the *IBM1* intron correlates with lower expression of its full-length isoform, suggesting how methylation status can distinctly influence IBM1’s functional role during different developmental phases. This complex regulatory mechanism demonstrates the crucial, tissue-specific role of epigenetic regulation in adapting to the developmental requirements of plants. Additionally, the rapid evolution observed in genes governing meiotic synapsis and recombination highlights the dynamic nature of genomic regulation by IBM1 [38]. The study shows IBM1’s involvement in meiotic chromosome synapsis and progression, suggesting its role as part of rapidly evolving genetic mechanisms that respond to evolutionary pressures. This complex regulatory mechanism not only illustrates the tissue-specific role of epigenetic regulation in plant development, but also highlights the evolving function of IBM1 in enhancing plant adaptability under diverse environmental conditions.

It’s unknown whether IBM1’s heterochromatin sensing ability extends outside of *A*. *thaliana*. DNA methylation of the intron within the JmjC domain of *IBM1* was common throughout flowering plants with notable exceptions in species that do not have gbM such as *E*. *salsugineum* and *T*. *arvense*. This could suggest that once gbM is lost there is no need for *IBM1* sensing activity, as ectopic H3K9me2/mCHG can no longer be recruited to these genes without CMT3 activity. We also identified numerous duplicate *IBM1* copies where one copy retained the methylated heterochromatin sensor intron and the other did not. This could indicate that the number of *IBM1* genes that possess the ability to sense heterochromatin is an important factor. Further exploration of the evolution of *IBM1* duplicates and the ability to sense heterochromatin via intron methylation will be needed to understand their role in maintaining proper gene expression and heterochromatin homeostasis. In addition, we observed that the sequence underlying the methylated heterochromatin sensor intron of *IBM1* was dynamically evolving between species even though heterochromatin sensor activity is likely retained. This suggests that there is a dynamic interplay between the evolution of heterochromatin abundance and the activity of IBM1 and CMT3. Identifying the triggers of this highly evolving process will be important for understanding how heterochromatin abundance is modified during flowering plant genome evolution.

It is curious that there are repeated occurrences of loss or reduced activity of CMT3 along with reduced or loss of gbM in some plant genomes. Why are there so many examples of deletion and/or loss of CMT3 activity? One possibility could be that there are genome-wide events that disrupt epigenome homeostasis, such that IBM1 no longer efficiently removes H3K9me2 from gbM genes. This would likely lead to a rapid loss of fitness due to decreased expression of many ‘housekeeping genes’. However, secondary site mutations in CMT3 would eliminate this silencing effect. We have identified numerous examples in this study where there is support for this model. For example, *E*. *salsugineum* has lost *CMT3* and has reduced expression of *IBM1*. We even identified a species that has lost *IBM1* (*I*. *lusitanica*). Even though *I*. *lusitanica* has *CMT3*, mCHG levels and symmetry analysis of mCHG shows that it is not functional supporting it is a natural double mutant of *IBM1* and *CMT3*. Higher quality genome sequence efforts will be required to confirm the loss of *IBM1*. Regardless, this species has lost CMT3 activity and gbM. Future studies are also needed to evaluate the extent to which *IBM1’s* heterochromatin sensor is functional across flowering plants. This is difficult to assay, given its discovery was dependent on the use of DNA methylation mutants in *A*. *thaliana*, which are not well tolerated in many flowering plants. Continual exploration of the evolution of *IBM1* and *CMT3* in newly released flowering plant genomes, especially within the Brassicaceae, will deepen our understanding of plant epigenome homeostasis.

## Methods

### WGBS and RNA-seq data acquisition

We obtained whole genome bisulfite sequencing (WGBS) and RNA-seq data for natural accessions of *A*. *thaliana* from the 1,001 Genomes Project [43–45,63]. The WGBS and RNA-seq data for *A*. *thaliana* mutants were obtained from NCBI [11,41,46–48], with specific details provided in S2 Table. Additionally, we acquired WGBS and RNA-seq data for 34 angiosperm species from NCBI [19,28,32,64–67], with the sources detailed in S7 Table. Furthermore, we generated WGBS and RNA-seq data for two Brassicaceae family species, and both the sequencing and processed data are available at NCBI GEO database (GSE252913).

### Plant materials and transgenic line analysis

The *IBM1* coding sequence from Col-0 was driven by the *UBQ10* promoter. The construct was transformed into the *A*. *thaliana* Cnt-1 accession by *Agrobacterium*-mediated flower dipping method [68]. Three transgenic lines were selected and RNA was extracted to estimate *IBM1* expression. The RT-qPCR analysis was conducted using the Luna Universal One-Step RT-qPCR Kit. The expression levels of *IBM1* were quantified and subsequently normalized against the expression levels of *UBQ10*. The genomic DNA from those lines were subjected to WGBS analysis. For the DNA methylation and RNA-seq analyses of the two Brassicaceae species, namely *Raphanus raphanistrum*, and *Isatis lusitanica*, the plants were grown under identical conditions to those used for *A*. *thaliana*. Leaf tissues were collected from each species at the 8-week-old growth stage for subsequent analyses.

### Whole genome bisulfite sequencing library preparation

Libraries were prepared following the MethylC-seq protocol [69]. Briefly, genomic DNA was isolated from leaf tissues using the DNeasy Plant Mini Kit (Qiagen). Subsequently, genomic DNA was sonicated to achieve 200 bp fragments, and then end-repair was performed by the End-It DNA End-Repair Kit (Epicentre). This end-repaired DNA was subjected to A-tailing using the Klenow 3′–5′ exo− enzyme (New England Biolabs). The subsequent step involved the ligation of methylated adapters to the A-tailed DNA, using T4 DNA Ligase (New England Biolabs). Following adapter ligation, the DNA was bisulfite converted with the EZ DNA Methylation-Gold Kit. Finally, the library was amplified using KAPA HiFi Uracil + Readymix Polymerase (Roche).

### DNA methylation analysis

WGBS data were processed using Methylpy [70], following the methodology outlined in reference [39]. Initially, read quality filtering and adapter trimming were conducted using Cutadapt v1.9.dev1. The qualified reads were then aligned to the species-specific reference genome using Bowtie 2.2.4 [71], ensuring that only uniquely aligned and non-clonal reads were retained. The genome assembly version of 34 species used for mapping are provided in S7 Table. To calculate the non-conversion rate of unmodified cytosines in the sodium bisulfite reaction, a fully unmethylated sequence, chloroplast or lambda (see unmethylated sequence used for each species in S7 Table), was used as a control. A binomial test, requiring a minimum coverage of three reads, was used to determine the DNA methylation status of cytosines.

To determine gbM genes in each of the 34 species, we counted the number of methylated and total cytosines for each methylation context (CG, CHG, and CHH) within the coding regions of primary transcripts for each gene. We then calculated the percentage of methylated sites for each context across all coding regions in each species. This percentage served as the background probability of methylation at a single site within coding sequences (CDS). Using this background probability, along with the total counts of cytosines and methylated cytosines on CDS, we calculated p-values based on a binomial distribution. These p-values represent the cumulative probability of observing a greater number of methylated cytosines in a given gene than expected by chance. Subsequently, we adjusted the p-values using the Benjamini–Hochberg False Discovery Rate (FDR) method to compute q-values. A gene was classified as having gbM if it had reads mapping to at least 20 CG sites, a q-value less than 0.05 for mCG, and q-values greater than 0.95 for both mCHG and mCHH. To calculate the gbM ratio, the total number of gbM genes was divided by the overall count of genes with adequate coverage in each species. This process of calculating gene coverage required counting CG sites within each gene, selecting those genes where at least 40% of the CG sites had a minimum coverage of three reads or more.

In natural *A*. *thaliana* accessions, we identified ectopic non-CG genes characterized by high non-CG methylation (mCHG and/or mCHH with a q-value less than 0.05) in certain accessions, while typically existing as gbM genes in over 90% of all accessions. Specifically, in Cnt-1, which has the highest number of ectopic mCHG in genes, we compared the mCHG levels of these genes between the wild type Cnt-1 and an *IBM1* overexpression transgenic line in Cnt-1 (*IBM1-ox*). The average DNA methylation ratio of each gene was calculated, and the number of the gbM genes that gained more than 2% mCHG (> 0.02) was plotted. The DNA methylation ratio of the mCHG-gain gbM genes in Cnt-1 (n = 636) were plotted as heatmaps.

Using binomial tests, we identified introns enriched with mCHG in *IBM1* of different species. For this, we counted the methylated and total cytosines for each methylation context (CG, CHG, and CHH) specifically within the 7th intron of *IBM1*. We then calculated the percentage of methylated sites for each context in all introns for each species. This species-specific percentage served as the background probability. With this background probability and the total counts of cytosines and methylated cytosines in the *IBM1* intron region, we computed p-values using a binomial distribution. These p-values were then adjusted using the Benjamini–Hochberg False Discovery Rate (FDR) method to derive q-values. An intron was classified as mCHG-enriched if its mCHG q-value was less than 0.05.

In the genic metaplots for *A*. *thaliana*, we divided the gene body into 20 equal windows. Similarly, the 1,000 base pairs upstream and downstream of the gene were each divided into 20 windows. Within each window, we calculated the weighted DNA methylation [72]. Subsequently, we computed the average weighted methylation for each window across all genes. These average values were then plotted using R to create the metaplots.

### Per site methylation and symmetry analysis

The methylation ratios at individual CHG sites were calculated under the condition that each CHG site exhibited a minimum read coverage of three. Additionally, a CHG site was included in the analysis only if at least one CHG site was identified as being methylated. For the analysis of methylation symmetry, we selectively focused on CWG sites (W = A or T) to exclude the influence of MET1’s activity on CCG sites. Both strands of CWG sites were required to have a minimum coverage of at least three reads, and at least one CWG site was identified as being methylated.

### Isoform quantification for RNA-seq data

Quality filtering and adapter trimming of the RNA-seq reads were conducted using Trimmomatic v0.33 [73], using default parameters. For each species, the processed reads were then aligned to their respective transcriptome fasta files using Kallisto v0.50.0 [74] for transcript quantification. Specifically in *A*. *thaliana*, to quantify the expression of short transcripts in genes with long introns, we generated truncated versions of these transcripts, comprising only the UTR and CDS regions preceding the long intron. These truncated transcripts were subsequently incorporated into the transcriptome files used for mapping. Similarly, for other Brassicaceae species with long introns in *IBM1*, we created and added shortened versions of *IBM1* transcripts into their respective transcriptome files for precise quantification of the short isoform expression of *IBM1*.

### Identification of *IBM1* and *CMT3* in the Brassicaceae species

The orthologs of *IBM1* and *CMT3* across four Brassicaceae species were identified using OrthoFinder [75] and the extracted coding sequences were used for phylogenetic analysis using MEGA [76]. The *de novo* transcriptome assembly was performed using Trinity [77]. The presence/absence of the *IBM1* and *CMT3* transcripts were identified by BLASTing the *A*. *thaliana* gene sequence against the assembled transcriptome. To amplify the *IBM1* and *CMT3* gene from the genomic DNA, the degenerate primers were designed (S11 Table), and the genes were PCR-amplified using Q5 High-Fidelity 2X Master Mix (New England Biolabs).

### Homologous genes identification and phylogeny analysis

We extracted the JmjC gene family from the One Thousand Plants (1KP) Consortium’s orthogroupings [78], using the *A*. *thaliana IBM1* gene identifier (AT3G07610). The 1KP Consortium identified a single orthogroup that encompasses the IBM1 proteins, along with three other JmjC family genes (JMJ27, JMJ26, JMJ29) from *A*. *thaliana*, with a total of 9,258 protein sequences. The corresponding coding sequences (CDS) for these genes were also obtained from the 1KP Consortium. We expanded our dataset to include sequences from 69 species not covered in 1KP, including 45 from the Brassicaceae family. Their annotated CDS and protein sequences were obtained from Phytozome or EBI, with specific data sources detailed in S6 Table. For these species not included in iKP orthogroup, protein sequences showing reciprocal best BLAST hits with *A*. *thaliana* JMJ27 (AT4G00990), JMJ26 (AT1G11950), JMJ29 (AT1G62310), and IBM1 (AT3G07610) were added in the orthogroup. In total, the JmjC gene family included 9,521 sequences from 1,130 species. Then, according to Interproscan, sequences were retained if they included the same PFAM domain (JmjC domain PF02373) or ProSiteProfiles domain (JmjC domain profile PS51184) as *A*. *thaliana*. These filtered sequences included 1,528 sequences from 602 species, from which 10 sequences were excluded due to discrepancies between their protein and CDS sequences in terms of codon-to-amino acid conversion.

Then, to estimate the JmjC gene tree, a protein alignment was carried out using Pasta [79] with the default setting. The resulting alignment was back-translated using the coding sequence into an in-frame codon alignment. Then, Gblocks was used to retain only conserved codons with default settings, but allowing for a 50% gapped position. This conserved codon alignment then served as the input for phylogenetic estimation using RAxML [80], which included 500 rapid bootstrap replicates. The generated tree was rooted at the green algae clade and subsequently edited using the R package ggtree. From this comprehensive gene tree, a subtree comprising *IBM1* orthologous genes from 34 species with available methylome data was extracted using ggtree.

### Identification of introns where decreased mCHG correlates with full-length transcripts in *A*. *thaliana*

For each gene featuring a long intron (≥1kb) enriched with mCHG, we determined the average expression of both short and long isoforms across replicates, conducting these calculations independently for wild-type and *suvh456* samples. Subsequently, we identified genes exhibiting a difference greater than 2 in the short-to-long isoform ratio between *suvh456* mutants and wild type.

### Calculation of SNP mutation rates

To determine the SNP density within the *A*. *thaliana* population, we divided the SNP genotype data of *IBM1* genes (sourced from the 1,001 Genomes Project) into three groups: SNPs in the CDS region, SNPs within the methylated 7th intron, and SNPs in other intron regions not covered by methylation. We then calculated the SNP density and Tajima’s D values separately for each of these SNP matrices. Tajima’s D values were calculated by R package PopGenome while SNP density was calculated as the proportion of sites with SNP in each of three groups.

To calculate the nucleotide mutation rate between *A*. *thaliana* and *A*. *lyrata*, we first generated separate sequence alignments for the coding sequences (CDS) and intron regions of both species. The intron regions were further categorized into methylated regions and non-methylated regions. For each of these three groups—CDS, methylated introns, and non-methylated introns—we counted the number of identical nucleotides shared between the two species. The nucleotide difference for each group was then determined by calculating 1 minus the proportion of identical bases.

### Annotation of repeats and motifs in *IBM1* introns across various species

The DNA sequences of the introns for each of the 34 species were extracted from their respective genome assemblies using GFF annotations. RepeatMasker [81] was then used to identify various types of transposable elements, utilizing Repbase [82] as the reference library.

## Supporting information

S1 Fig Features related to *IBM1* intronic methylation in *A*. *thaliana* natural accessions. (PDF)

S2 Fig Features related to *IBM1* intronic methylation in *A*. *thaliana* mutant lines. (PDF)

S3 Fig DNA methylation analysis in the *IBM1*-ox lines within the Cnt-1 background. (PDF)

S4 Fig Phylogenetic relationships of JmjC family homologous genes across plants. (PDF)

S5 Fig A complete version of the maximum likelihood gene tree that includes 65 *IBM1* orthologs. (PDF)

S6 Fig Identification of Brassicaceae species with potential loss or truncations of *IBM1* or *CMT3*. (PDF)

S7 Fig The expression levels of *CMT1*, *CMT2*, *CMT3*, *IBM1* and *KYP* in Brassicaceae species. (PDF)

S8 Fig DNA methylation analysis in Brassicaceae species. (PDF)

S9 Fig CWG methylation symmetry analysis in Brassicaceae species. (PDF)

S1 Table Reads count on the 7th intron of *IBM1*. (XLSX)

S2 Table RNA-seq data source of DNA methylation mutant lines. (XLSX)

S3 Table Methylation distribution in introns larger than 1kb in *A*. *thaliana*. (XLSX)

S4 Table The short and long isoform expression for genes with large introns. (XLSX)

S5 Table Mutation rate in different regions of *IBM1* gene among nature *A*.*thaliana* population and at species level. (XLSX)

S6 Table JmjC family homologous genes from different species. (XLSX)

S7 Table Data source and genome information used in the DNA methylation analysis. (XLSX)

S8 Table DNA methylation and TE status in introns of 34 species. (XLSX)

S9 Table Blast results between the 7th intron of *Arabidopsis thaliana* and introns from IBM1 orthologs of the 34 species. (XLSX)

S10 Table Expression of *IBM1*, *CMTs* and *KYP* in 34 species. (XLSX)

S11 Table Primers used in this study. (XLSX)

S12 Table The CMT3 sequences of *I*.*lusitanica* and *R*.*raphanistrum*. (XLSX)
