# Supplementary material for: Dynamic evolution of the heterochromatin sensing histone demethylase IBM1
Source: PLoS Genet. 2024 Jul 11;20(7):e1011358. doi: 10.1371/journal.pgen.1011358 (PMC11265718; doi:10.1371/journal.pgen.1011358)
Supplement: S1 Fig — (PDF) [file pgen.1011358.s001.pdf]

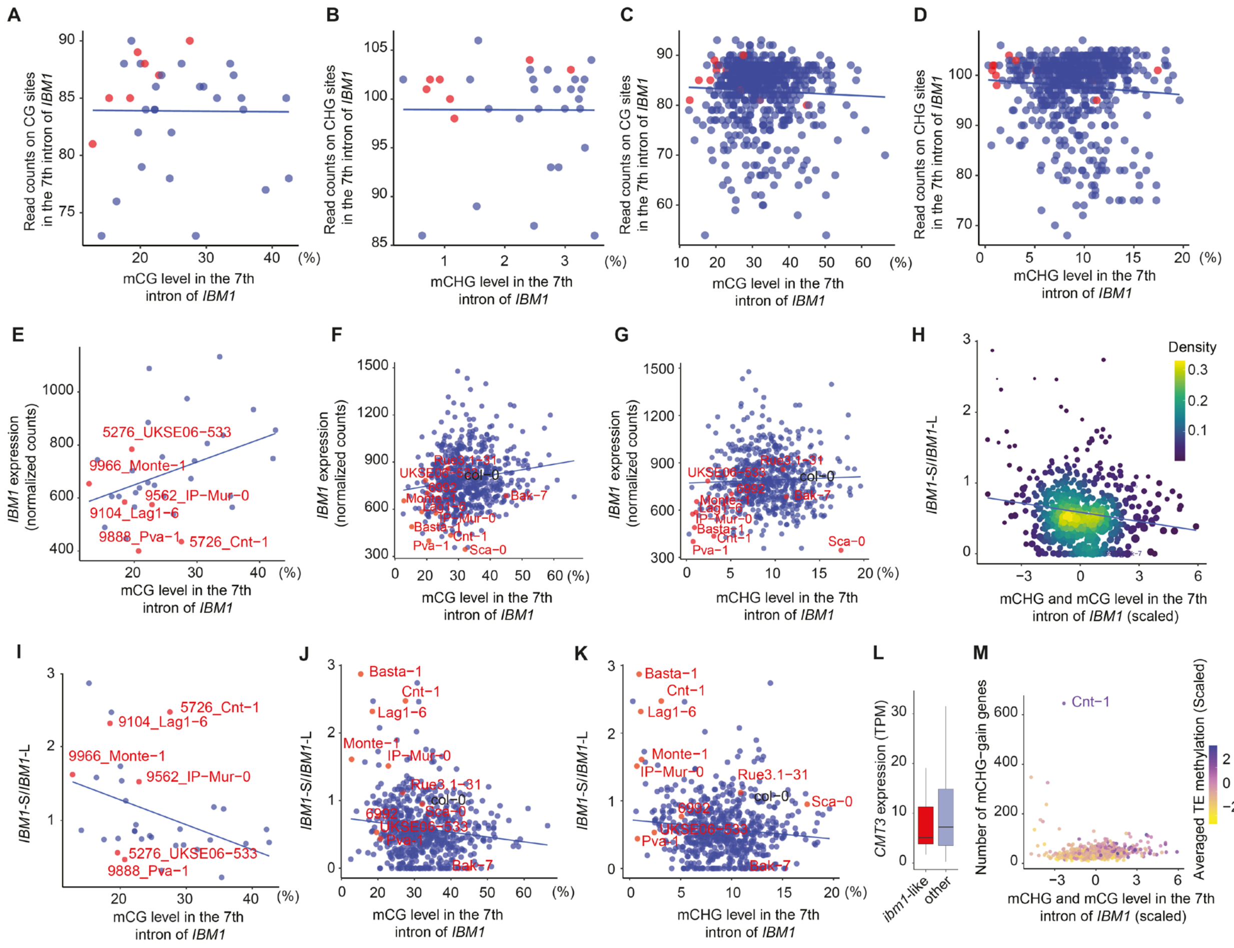

S1 Fig. Features related to *IBM1* intronic methylation in *A. thaliana* natural accessions. (A) Scatter plots (A-D) show the read counts on the *IBM1* intron region against the intron DNA methylation level of the long intron of *IBM1*. This plot shows the read counts on the CG sites of the *IBM1* intron region against the CG methylation level of the long intron of *IBM1*. Only accessions with mCHG level <0.035 in the long intron of *IBM1* were included. Accessions with a significant number (n>120) of ectopic methylated genes are marked with red dots, whereas other accessions are represented with blue dots. **(B)** Similar to A, except for the read count on the CHG sites against the CHG methylation level. **(C)** Similar to A, except for including all natural accessions. **(D)** Similar to B, except for including all natural accessions. **(E)** The scatter plot shows the relationship between CG methylation levels in the long intron of *IBM1* and *IBM1* gene expression for accessions with low *IBM1* long intron mCHG level (<= 0.035). The accessions with the highest number of ectopic methylated genes are labeled by their name in red color. **(F-G)** The scatter plot presents the (F) CG and (G) CHG methylation levels in the long intron of *IBM1* plotted against the *IBM1* expression level for all natural accessions. **(H)** The scatter plot presents the DNA methylation level in the long intron of *IBM1* plotted against the ratio of short to long isoform of *IBM1* for all natural accessions. The x-axis represents the cumulative value of scaled CG and CHG methylation levels. **(I)** Similar to E, this scatter plot presents the CG methylation level in the long intron of *IBM1* plotted against the ratio of short to long isoform of *IBM1*. (**J-K)** The scatter plot presents the (J) CG and (K) CHG methylation levels in the long intron of *IBM1* plotted against the ratio of short to long isoform of *IBM1* for all natural accessions. **(L)** The box plot shows the distribution of *CMT3* gene expression by comparing

accessions that rank in the top 11 for the highest number of ectopic methylated genes with all remaining accessions. **(M)** The scatter plot shows the scaled CG and CHG methylation level in *IBM1's* long intron against the number of genes with ectopic non-CG methylation in each natural accession. Accession Cnt-1 with the highest number (n=646) of ectopic methylated genes is labeled by its name. Each dot represents an accession and is colored by their averaged TE methylation level across the whole genome.
