# Supplementary material for: Dynamic evolution of the heterochromatin sensing histone demethylase IBM1
Source: PLoS Genet. 2024 Jul 11;20(7):e1011358. doi: 10.1371/journal.pgen.1011358 (PMC11265718; doi:10.1371/journal.pgen.1011358)
Supplement: S2 Fig — (PDF) [file pgen.1011358.s002.pdf]

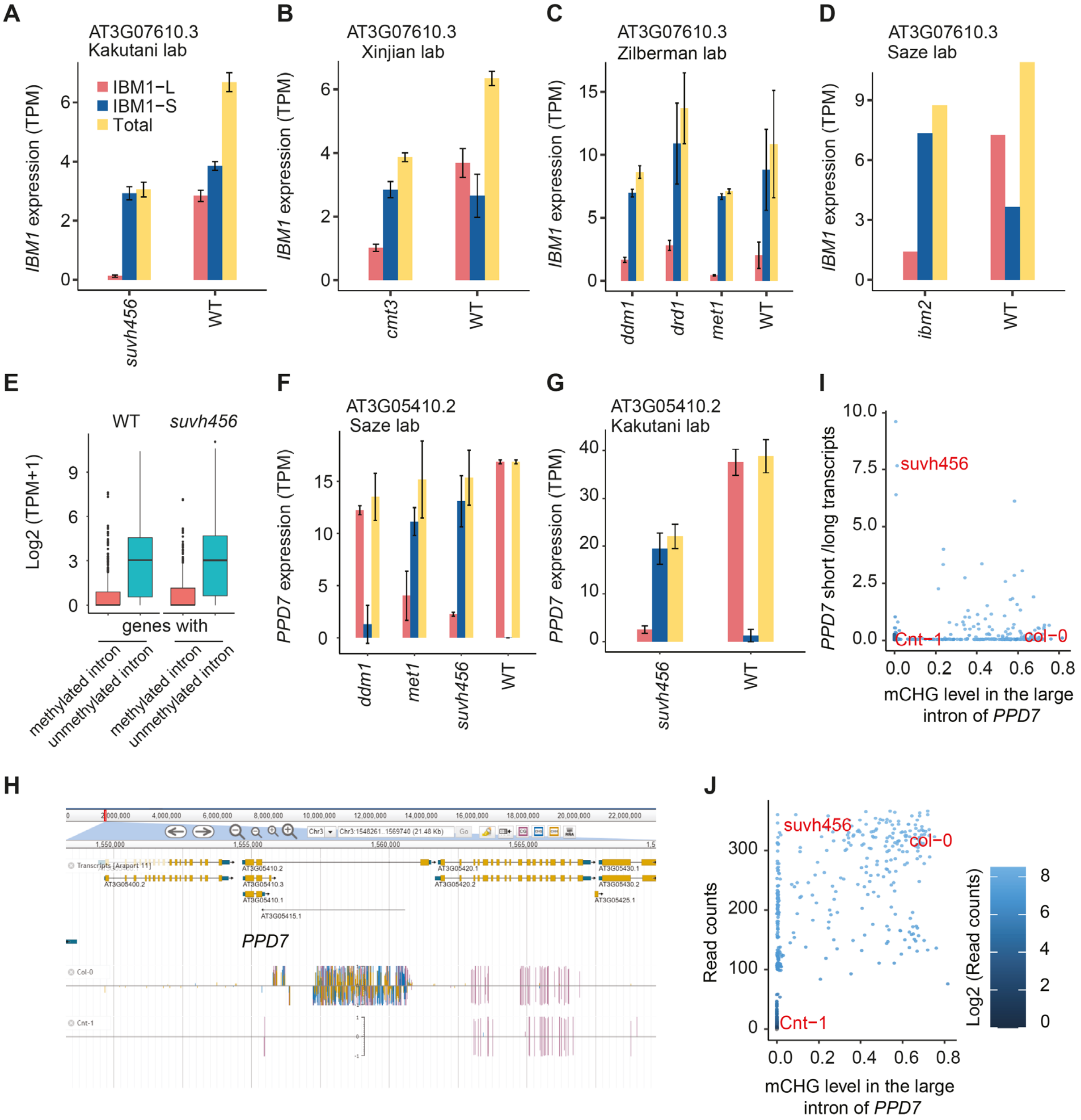

S2 Fig. Features related to *IBM1* intronic methylation in *A. thaliana* mutant lines. (A) The bar plot shows the expression levels of the long and short isoforms of *IBM1*, as well as their combined expression, in *suvh456* mutants, and Col-0 WT using RNA-seq data sourced from the Kakutani lab. **(B)** Jianxi lab, **(C)** Zilberman lab, **(D)** Plant epigenetics unit. **(E)** Genes featuring long introns (≥1kb) were categorized into two groups based on the presence or absence of CHG methylation. The box plot compares the expression distribution between these two groups. It displays the expression patterns of genes in both the wild type (right panel) and suvh456 mutant (left panel). **(F)** the long and short isoforms of *PPD7* using RNA-seq data sourced from Saze lab, and **(G)** Kakutani lab. **(H)** A genome browser view shows the intron methylation pattern of *PPD7* in Col-0, which is lost in Cnt-1. **(I)** The scatter plot shows the relationship between mCHG levels in the long intron of *PPD7* against the ratio of short to long isoform of *PPD7* for

all natural accessions. The *suvh456* mutant, Cnt-1 and Col-0 are highlighted by their name in red color, while other accessions are represented by blue dots. **(J)** The scatter plot shows the relationship between CHG methylation levels in the long intron of *PPD7* against the read counts on the CHG sites of long intron of *PPD7*. The color scheme is the same as H.
