# Supplementary material for: Dynamic evolution of the heterochromatin sensing histone demethylase IBM1
Source: PLoS Genet. 2024 Jul 11;20(7):e1011358. doi: 10.1371/journal.pgen.1011358 (PMC11265718; doi:10.1371/journal.pgen.1011358)
Supplement: S3 Fig — (PDF) [file pgen.1011358.s003.pdf]

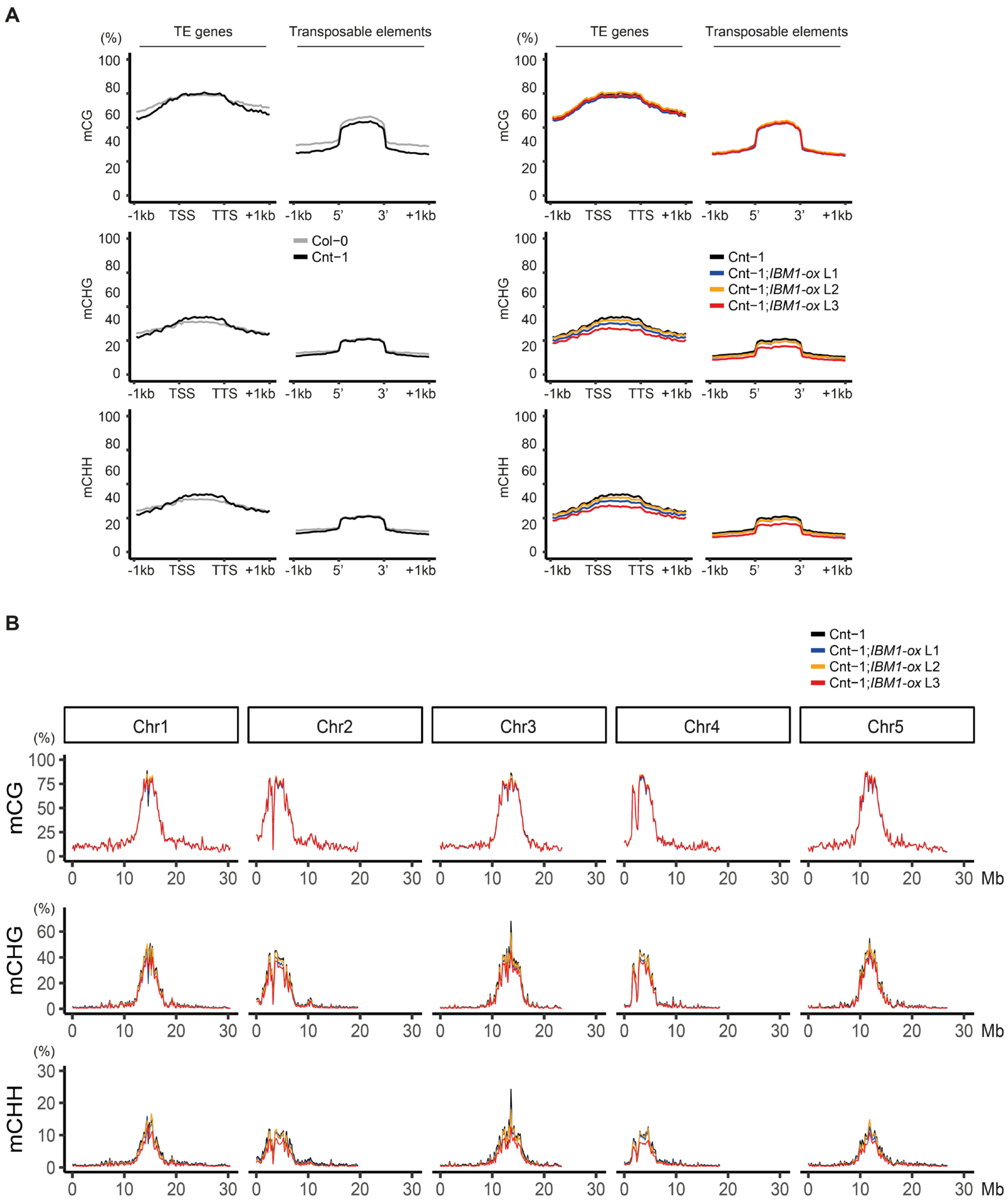

S3 Fig. DNA methylation analysis in the *IBM1*-ox lines within the Cnt-1 background. (A) Metaplots of DNA methylation over transposable element (TE) genes and transposable elements (TEs) in the *IBM1-ox* lines within the Cnt-1 background, compared to Col-0. Methylation levels are shown for CG, CHG, and CHH contexts. **(B)** Chromosomal plots of DNA methylation in the

IBM1-ox lines within the Cnt-1 background. The plots display methylation levels across chromosomes 1 to 5 for CG, CHG, and CHH contexts.
