# Supplementary material for: Dynamic evolution of the heterochromatin sensing histone demethylase IBM1
Source: PLoS Genet. 2024 Jul 11;20(7):e1011358. doi: 10.1371/journal.pgen.1011358 (PMC11265718; doi:10.1371/journal.pgen.1011358)
Supplement: S4 Fig — (PDF) [file pgen.1011358.s004.pdf]

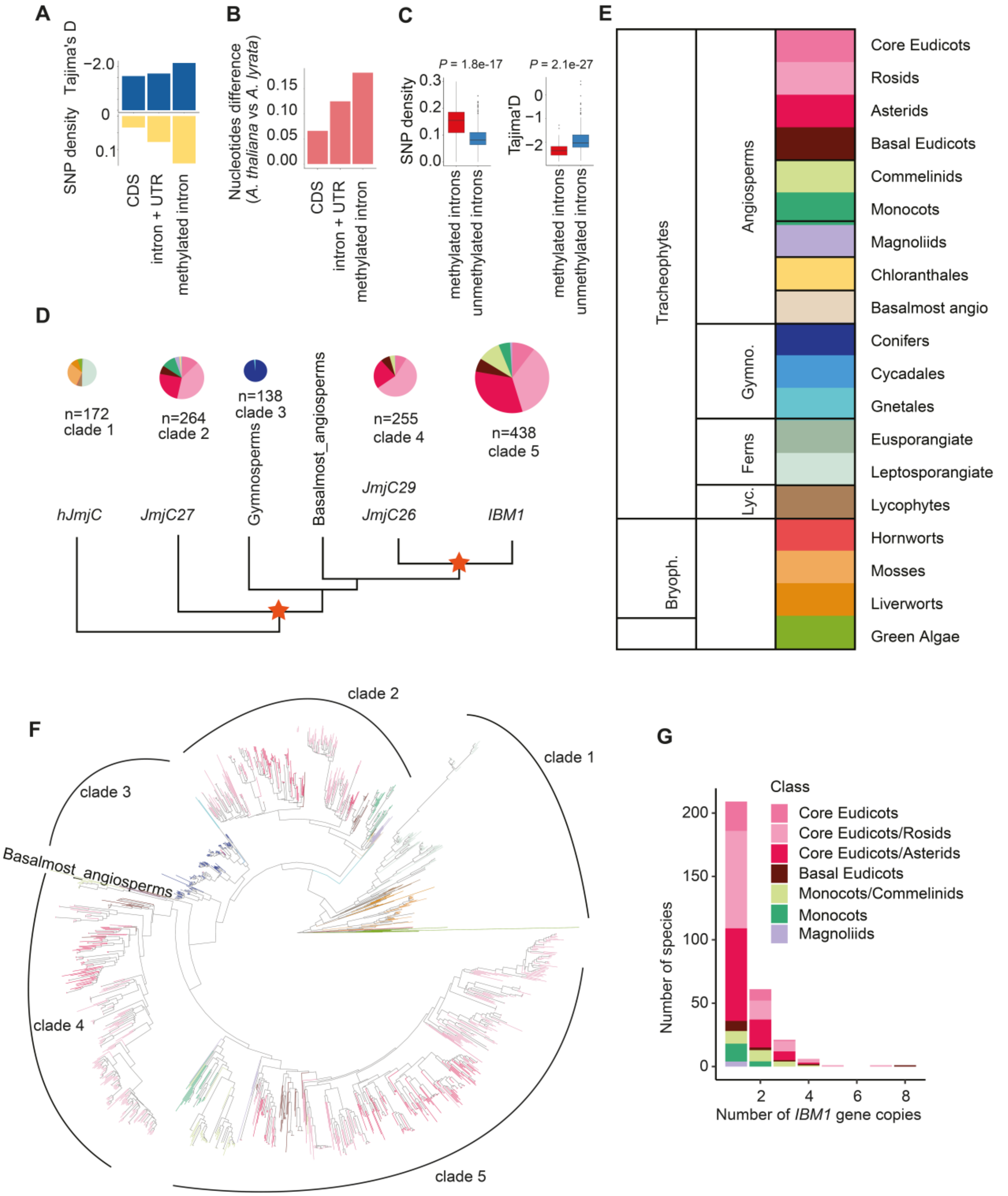

S4 Fig. Phylogenetic relationships of JmjC family homologous genes across plants. (A) The plot shows Tajima's D and SNP density for *IBM1*, comparing its coding sequence (CDS), noncoding regions (inclusive of UTRs and introns not encompassed by non-CG methylation), and the region of methylated introns. The SNP data for this analysis was sourced from the 1,001 Genomes Project. **(B)** The plot illustrates the nucleotide differences, calculated as the proportion

of mismatched bases, between *A. thaliana* and *A. lyrata*. These differences are shown separately for the DNA sequences in the coding sequence (CDS), non-coding regions, and the methylated intron region. **(C)** The boxplot on the left illustrates the distribution of SNP density in long introns (≥1kb), categorized into two groups based on the presence or absence of CHG methylation. Similarly, the plot on the right panel compares Tajima's D between these two groups. **(D)** The gene family tree of the JmjC domain is presented as a collapsed version, delineating six distinct subclades (also described in E). Pie charts, scaled according to the number of species, depict the species diversity within each clade. Two significant duplication events are highlighted: one shared by all angiosperms and gymnosperms, and another exclusive to all angiosperms except for the basalmost group (indicated by a star symbol). These events led to the diversification of various JmjC homologous genes, such as *JmjC27*, *JmjC26/29*, and *IBM1*. The tree is rooted in the clade that encompasses all green algae and liverwort species. **(E)** The plot provides the color scheme for species classification as used in Fig. S3. **(F)** The circular gene tree illustrates JmjC homologous genes organized into six distinct clades (the same as C), following the relationships of *A. thaliana* JmjC genes. These clades include: (1) homologous JmjC genes in green algae, bryophytes, and ferns; (2) *JmjC2*7; (3) JmjC genes in gymnosperms; (4) JmjC genes in basalmost angiosperms; (5) *JmjC26/29*; and (6) *IBM1*. Notably, the *JmjC26/29* and *IBM1* clades encompass all angiosperm species, with the exception of the basalmost angiosperms. This distribution is due to a duplication event that occurred just prior to the divergence of other angiosperms. **(G)** The bar plot displays the distribution of the number of *IBM1* gene copies across all species within the *IBM1* gene clade.
