# Supplementary material for: Dynamic evolution of the heterochromatin sensing histone demethylase IBM1
Source: PLoS Genet. 2024 Jul 11;20(7):e1011358. doi: 10.1371/journal.pgen.1011358 (PMC11265718; doi:10.1371/journal.pgen.1011358)
Supplement: S5 Fig — (PDF) [file pgen.1011358.s005.pdf]

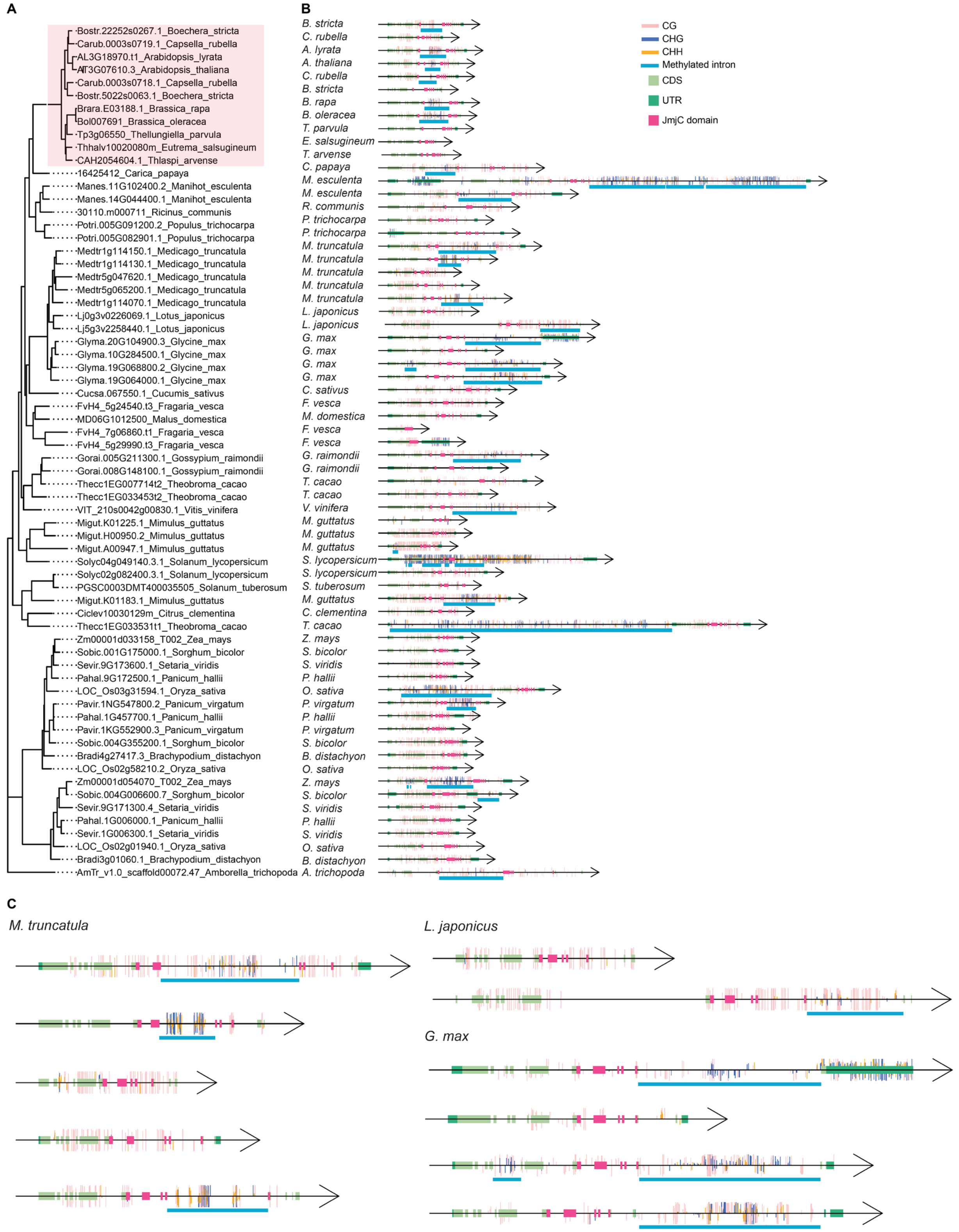

S5 Fig. A complete version of the maximum likelihood gene tree that includes 65 *IBM1* orthologs. The gene tree has the same format and color scheme as Fig 3A.
