# Supplementary material for: Dynamic evolution of the heterochromatin sensing histone demethylase IBM1
Source: PLoS Genet. 2024 Jul 11;20(7):e1011358. doi: 10.1371/journal.pgen.1011358 (PMC11265718; doi:10.1371/journal.pgen.1011358)
Supplement: S6 Fig — (PDF) [file pgen.1011358.s006.pdf]

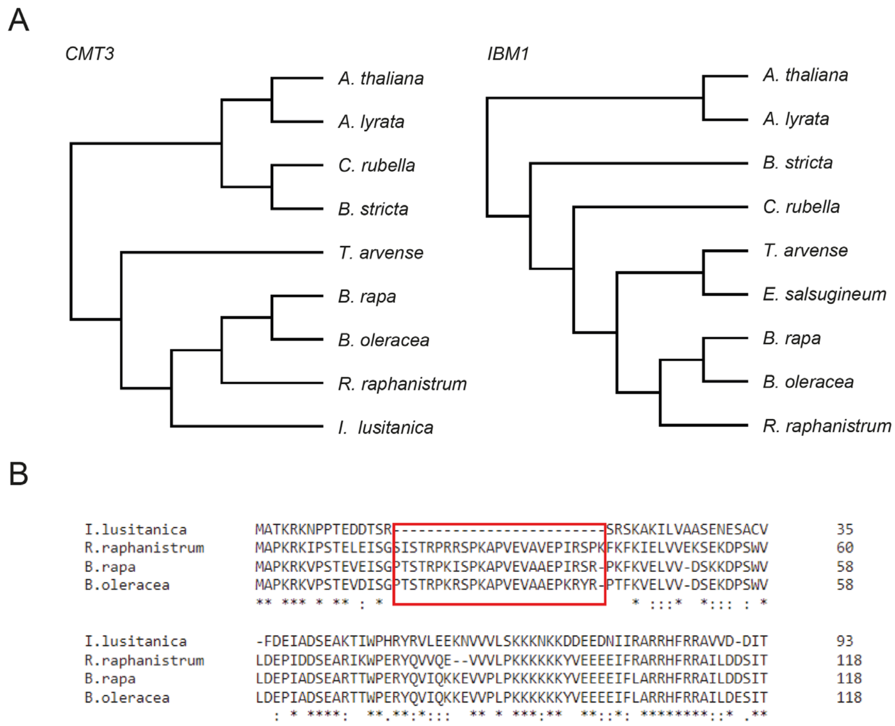

S6 Fig. Identification of Brassicaceae species with potential loss or truncations of *IBM1* or *CMT3*. **(A)** Ortholog analysis of *CMT3* and *IBM1* in the Brassicaceae species. **(B)** An alignment showing the N-terminal deletion in CMT3 of *I. lusitanica*.
