# Supplementary figures and images for: Dynamic evolution of the heterochromatin sensing histone demethylase IBM1
Source: PLoS Genet. 2024 Jul 11;20(7):e1011358. doi: 10.1371/journal.pgen.1011358 (PMC11265718; doi:10.1371/journal.pgen.1011358)

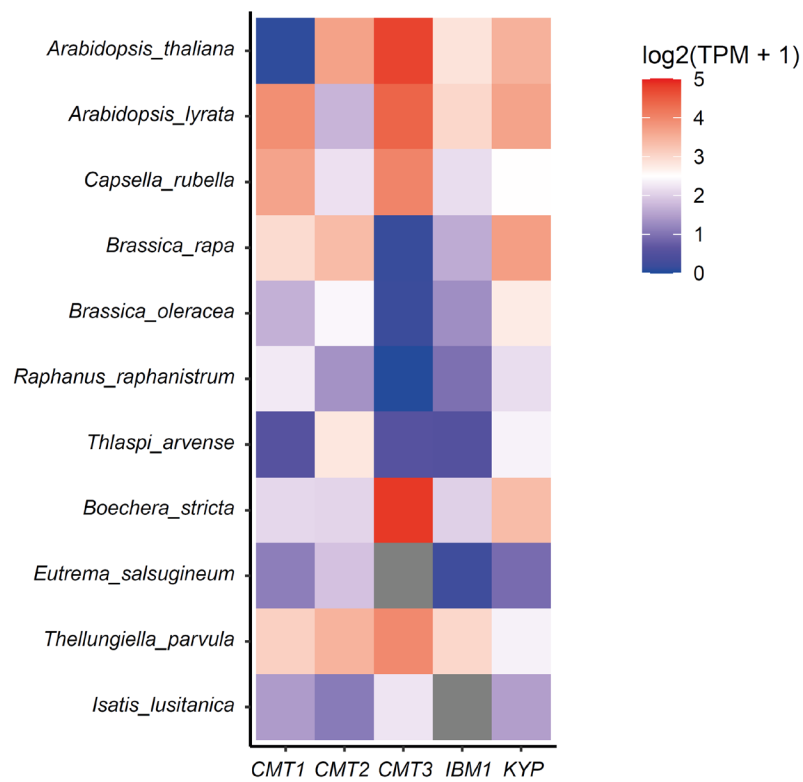

**S7 Fig.** The expression levels of *CMT1*, *CMT2*, *CMT3*, *IBM1* and *KYP* in Brassicaceae species.

Supplement: S7 Fig — (PDF) [file pgen.1011358.s007.pdf]
