# Supplementary material for: Dynamic evolution of the heterochromatin sensing histone demethylase IBM1
Source: PLoS Genet. 2024 Jul 11;20(7):e1011358. doi: 10.1371/journal.pgen.1011358 (PMC11265718; doi:10.1371/journal.pgen.1011358)
Supplement: S8 Fig — (PDF) [file pgen.1011358.s008.pdf]

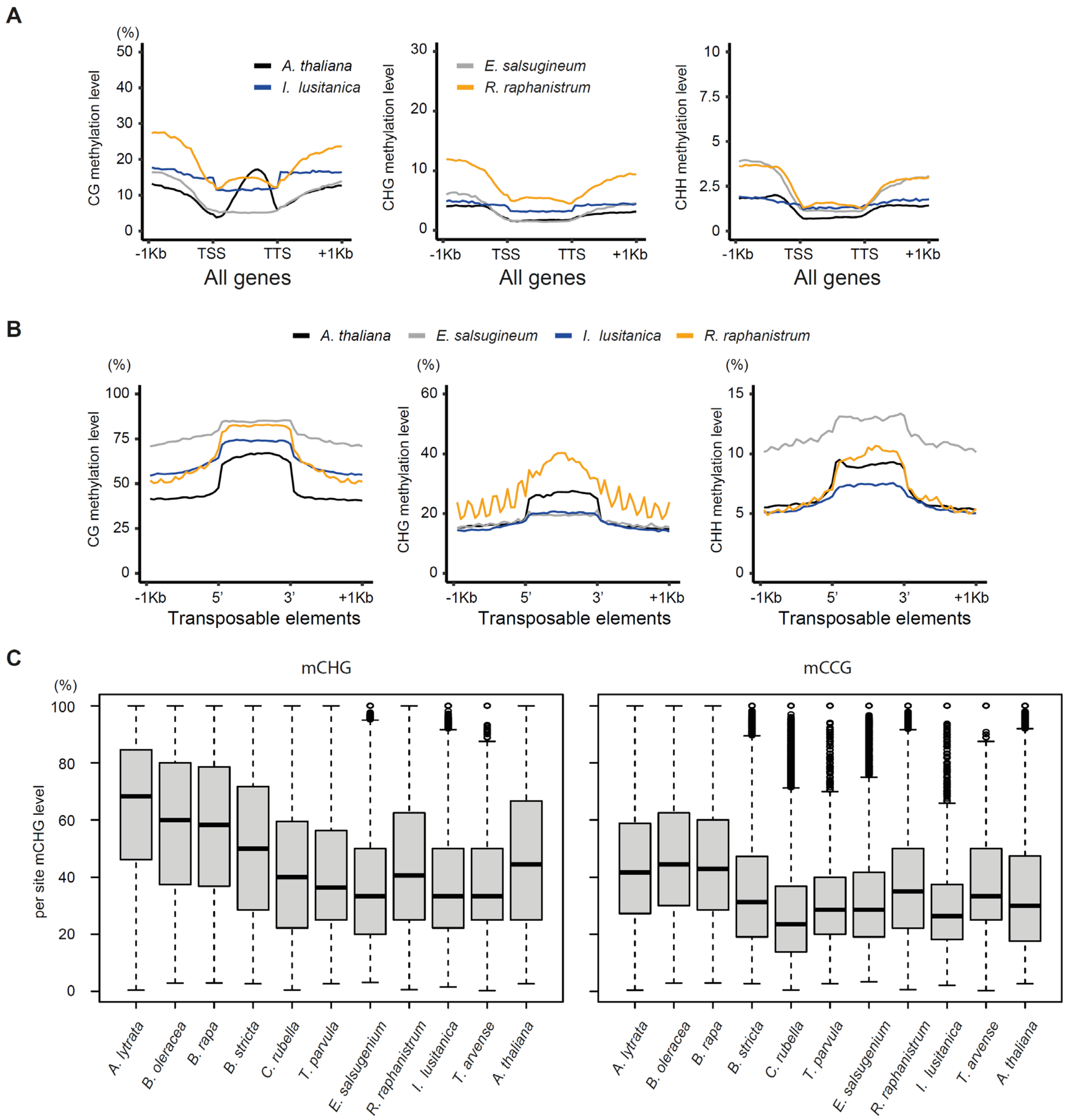

S8 Fig. DNA methylation analysis in Brassicaceae species. (A) Metaplots of DNA methylation levels across all sequence contexts (CG, CHG, CHH) over all genes in four Brassicaceae species. (B) Metaplots showing DNA methylation levels across transposable elements (TEs) in the same species. (C) Comparison of per-site methylation levels of CHG and mCCG sites in the Brassicaceae species.
