# Supplementary material for: Dynamic evolution of the heterochromatin sensing histone demethylase IBM1
Source: PLoS Genet. 2024 Jul 11;20(7):e1011358. doi: 10.1371/journal.pgen.1011358 (PMC11265718; doi:10.1371/journal.pgen.1011358)
Supplement: S9 Fig — (PDF) [file pgen.1011358.s009.pdf]

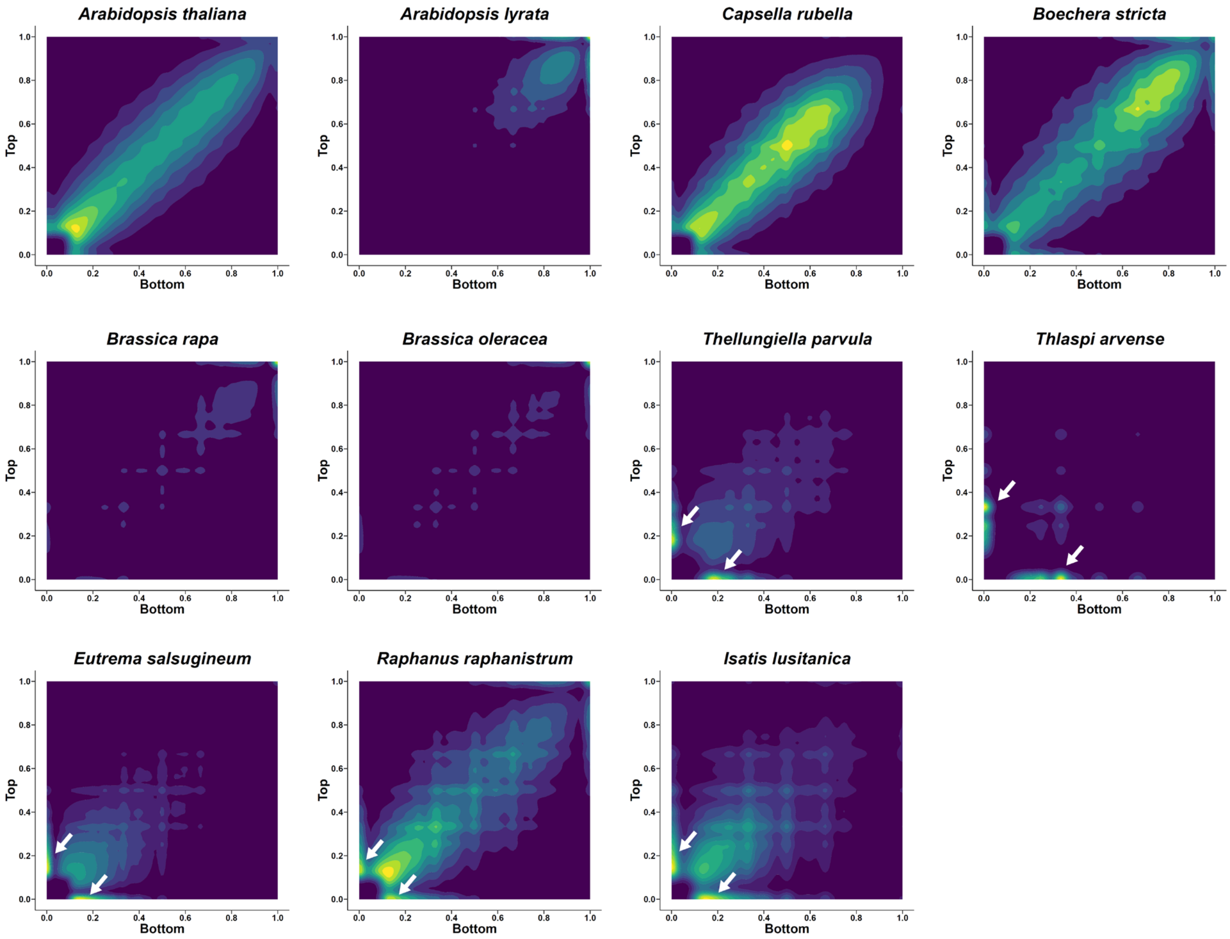

S9 Fig. CWG methylation symmetry analysis in Brassicaceae species. The arrows indicate asymmetry of CWG methylation.
